# Reverse engineering of the pattern recognition receptor FLS2 reveals key design principles of broader recognition spectra against evading flg22 epitopes

**DOI:** 10.1038/s41477-025-02050-5

**Published:** 2025-07-28

**Authors:** Songyuan Zhang, Songyuan Liu, Hung-Fei Lai, Kyle W. Bender, Gijeong Kim, Amedeo Caflisch, Cyril Zipfel

**Affiliations:** 1 Institute of Plant and Microbial Biology, Zurich-Basel Plant Science Center, University of Zurich, Zurich, Switzerland; 2 Department of Biochemistry, University of Zurich, Zurich, Switzerland; 3 The Sainsbury Laboratory, University of East Anglia, Norwich Research Park, Norwich, UK

**Keywords:** Pattern recognition receptors in plants, Plant signalling, Molecular engineering in plants

## Abstract

In the ongoing plant–pathogen arms race, plants use pattern recognition receptors (PRRs) to recognize pathogen-associated molecular patterns (PAMPs), while in successful pathogens, PAMPs can evolve to evade detection. Engineering PRRs to recognize evading PAMPs could potentially generate broad-spectrum and durable disease resistance. Here we reverse-engineered two natural variants of the PRR FLAGELLIN SENSING 2 (FLS2), VrFLS2XL and GmFLS2b, with extended recognition specificities towards evading flg22 variants. We identified minimal gain-of-function residues enabling blind FLS2s to recognize otherwise evading flg22 variants. We uncovered two strategies: (1) optimizing FLS2–flg22 interaction around flg22’s key evasion sites and (2) strengthening direct FLS2–BAK1 interaction to overcome weak agonistic and antagonistic flg22s, respectively. In addition, we leveraged polymorphisms that enhance recognition through unknown mechanisms to engineer a superior recognition capability. These findings offer basic design principles to engineer PRRs with broader recognition spectra, paving the way for PRR engineering to generate precisely gene-edited disease-resistant crops.

## Abstract (web summary)

By dissecting natural variants, Zhang et al. uncover design principles for engineering the FLS2 immune receptor with broad recognition spectra to detect evasive bacterial pathogens, offering a promising strategy to improve crop disease resistance.

## Main

As the first layer of plant immune recognition, plants use plasma membrane-localized pattern recognition receptors (PRRs) to detect microbe-associated and/or pathogen-associated molecular patterns (PAMPs) of potential invaders and initiate pattern-triggered immunity. One of the best-studied plant PRRs is the leucine-rich repeat (LRR) receptor kinase FLAGELLIN SENSING 2 (FLS2), which, along with the co-receptor BRASSINOSTEROID INSENSITIVE 1-ASSOCIATED KINASE 1 (BAK1), recognizes flg22 (a 22-amino-acid epitope derived from the N terminus of bacterial flagellin)^1–3^. FLS2 is ubiquitously present in angiosperms^4^ and plays a crucial role in bacterial disease resistance^5^.

Structural characterization of the FLS2–flg22–BAK1 complex^6^ and previous genetic and biochemical studies^2,7^ suggest a sequential ligand-induced heterodimerization mechanism of flg22 perception and receptor activation^8^. The flg22 peptide adopts an extended conformation to bind to the concave surface of FLS2’s extracellular LRR domain. Its N-terminal 17 amino acids (‘address’ segment) interact solely with FLS2, and its C-terminal 5 amino acids (‘message’ segment) together with FLS2 create a flg22-mediated FLS2–BAK1 interaction interface to recruit BAK1. The FLS2–flg22–BAK1 complex is further stabilized by another direct FLS2–BAK1 interaction interface independent of flg22. The heterodimerization of extracellular domains brings the intracellular kinase domains of FLS2 and BAK1 into proximity to trans-phosphorylate each other and activate the downstream signalling pathways^2,6,9,10^, such as reactive oxygen species (ROS) production, calcium influx, mitogen-activated protein kinase (MAPK) cascades, transcriptional reprogramming and callose deposition^11,12^.

PRR-mediated PAMP recognition imposes strong selective pressures on pathogens, driving them to evade the recognition^13,14^. For example, many bacterial phytopathogens carry polymorphic flg22 epitopes not recognizable by FLS2^15^. This includes *Agrobacterium tumefaciens*, the causative agent of crown gall disease^3,16^; *Xanthomonas oryzae* pv. *oryzae* and pv. *oryzicola*, which cause bacterial blight and leaf streak in rice^17^; *Xanthomonas campestris* pv. *campestris*, responsible for black rot in crucifers^18^; the bacterial wilt disease pathogen *Ralstonia solanacearum*^19^; and *Erwinia amylovora* that causes fire blight^20^. Such immune evasion is also common among the commensal microbiota of healthy plants^21–23^. Polymorphic flg22 variants apply diverse mechanisms to evade recognition: some act as weak agonists with reduced binding affinity to FLS2, while some act as antagonists that retain interaction with FLS2 but harbour C-terminal mutations to impede BAK1 recruitment. In addition, another three types of flg22 variant have been reported with atypical evasion behaviour through uncharacterized mechanisms^21^.

However, the evolution of non-immunogenic flg22 variants by pathogens can be countered by the co-evolution of FLS2 variants with novel recognition specificities. For example, VrFLS2XL from riverbank grapes (*Vitis riparia*, Vr) can recognize *A. tumefaciens* flg22 (flg22^Atum^)^24^, and GmFLS2b of soybean (*Glycine max*, Gm) can recognize *R. solanacearum* flg22 (flg22^Rso^)^25^. Recently, the generation of *Nicotiana benthamiana fls2* mutant has facilitated the rapid characterization of natural FLS2 variants, expanding the known repertoire of FLS2s with novel recognition specificities^26^. For example, QvFLS2 of *Quercus variabilis* (Qv) and TjFLS2 of *Trachelospermum jasminoides* (Tj) are also highly sensitive to flg22^Atum^ when transiently expressed in *N. benthamiana fls2* mutant^26^.

The interfamily transfer of PRRs has proven to be an effective strategy to generate broad-spectrum and durable disease resistance^27–29^, which holds true not only for phylogenetically restricted PRRs, like the ELONGATION FACTOR TU RECEPTOR, but also for the prevalent FLS2. For example, transferring VrFLS2XL to *N. benthamiana* and GmFLS2b to tomato conferred resistance to *A. tumefaciens* and *R. solanacearum*, respectively^24,25^. However, the broad applicability of PRR transfer is currently limited by the lack of known PRRs, difficulties in identifying new PRRs, the potential for pathogens to evolve evasion and legal restrictions on the use of transgenic plants.

A promising alternative is the use of precise gene-editing techniques to modify native PRRs, thereby expanding their recognition spectra^30^. The increasingly wider acceptance of genome-edited crops^31^ and the development of CRISPR (clustered regularly interspaced short palindromic repeats)-mediated precise genome-editing technologies^32^ can enable the fast deployment of engineered PRRs from the lab to the field. However, this approach is currently hindered by our limited knowledge of how to design PRRs with novel recognition specificities. While several previous studies have tried to understand novel specificities of FLS2s, either they did not generate a gain-of-function FLS2^25^ or the resolution of the mapped region is not fine enough to guide precise engineering^20,24,33^.

In this Article, we explored the basic engineering principles behind novel recognition specificities by reverse-engineering two natural FLS2 variants, VrFLS2XL and GmFLS2b. Using a combination of domain swapping, structure-guided mutagenesis and DNA shuffling, we identified minimal residues required to enable blind VvFLS2 of cultivated grape (*Vitis vinifera*) and GmFLS2a (an ortholog of GmFLS2b insensitive to flg22^Rso^) to recognize flg22^Atum^ and flg22^Rso^, respectively. Our findings revealed two key engineering principles: (1) optimizing FLS2–flg22 interaction around flg22’s key evading mutations to overcome weak agonistic flg22 variants and (2) strengthening direct FLS2–BAK1 interaction to overcome antagonistic flg22 variants. In addition to identifying minimal gain-of-recognition residues, we discovered polymorphic residues outside the flg22- and BAK1-interacting interfaces of GmFLS2b, which influence recognition specificity through unknown mechanisms. By leveraging these residues, we engineered a GmFLS2 variant exhibiting superior flg22^Rso^ recognition compared to GmFLS2b. Together with the accompanying study by Li et al.^34^, this study advances our understanding on the recognition specificity of FLS2 and reveals fundamental design principles to engineer broader recognition spectra. In addition, this study provides valuable genetic resources to generate crown gall and bacterial wilt disease resistance in economically important crops.

## Results

### LRR12-19^VrFLS2XL^ is crucial for flg22^Atum^ recognition

**Extended Data Fig. 1:**
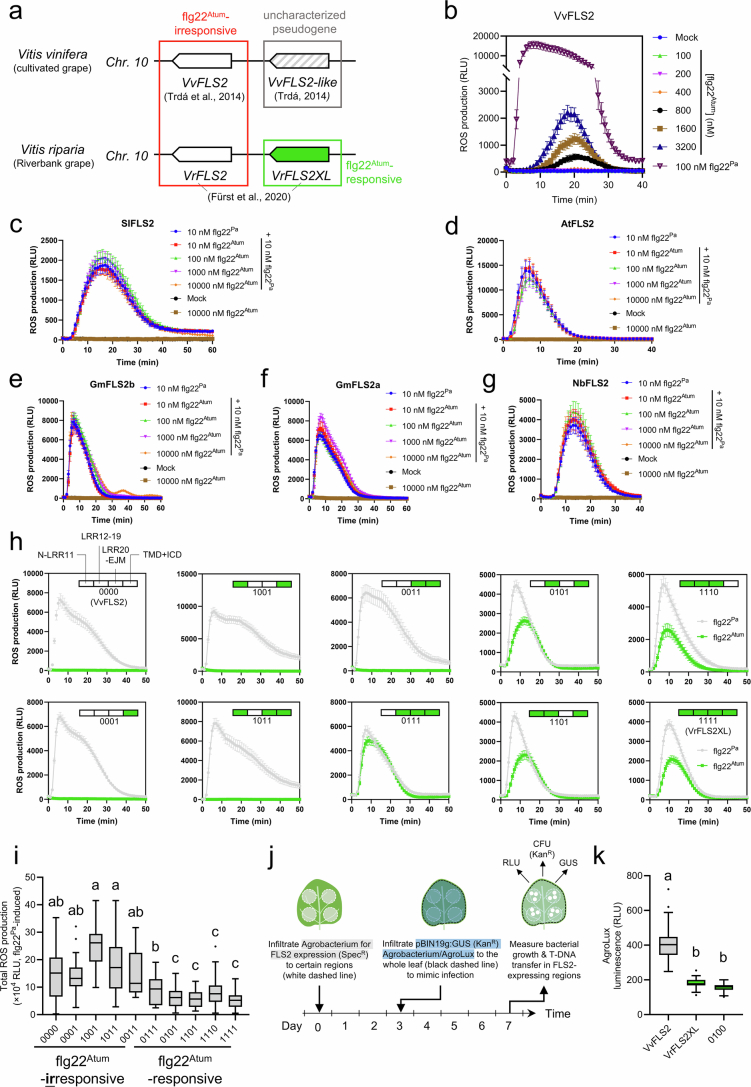
Evasion of recognition by flg22^Atum^ across different FLS2 homologs and flg22^Atum^ responsiveness of VvFLS2-VrFLS2XL chimeras. **a**, Summary of the reported flg22^Atum^ responsiveness of FLS2 homologs in the chromosome 10 (Chr. 10) of *Vitis vinifera* and *Vitis riparia*. **b**, Kinetics of VvFLS2’s ROS responses to flg22^Pa^ and increasing concentrations of flg22^Atum^ (related to Fig. 1b, *n* = 8 leaf discs). **c**–**g**, Characterization of flg22^Atum^ evasion and its potential antagonistic effect on SlFLS2 (**c**, *n* = 16 leaf discs), AtFLS2 (**d**, *n* = 24 leaf discs), GmFLS2b (**e**, *n* = 16 leaf discs), GmFLS2a (**f**, *n* = 16 leaf discs), NbFLS2 (**g**, *n* = 24 leaf discs). No significant differences in total ROS production were observed between treatments in panels **c**–**g**. **h**, ROS burst kinetics of chimeric receptors in response to flg22^Pa^ and flg22^Atum^ (related to Fig. 1d, *n* = 36, 52, 16, 28, 16, 40, 76, 48, 36, 64 leaf discs, from left to right). **i**, Total flg22^Pa^-induced ROS production of chimeric receptors (with the same sample sizes (*n*) as Fig. 1d). **j**, Experimental procedure used to assess the resistance to *A. tumefaciens* conferred by FLS2 variants. **k**, Significant restriction of *A. tumefaciens* growth by VrFLS2XL and ‘0100’ compared to VvFLS2 (*n* = 20 leaf discs). For line charts, data are shown as mean ± SEM. For box plots: center line, median; box limits, upper and lower quartiles; whiskers, 1.5 times interquartile range; points, outliers. Kruskal-Wallis test followed by Dunn’s multiple comparisons test was used for statistical analysis in panels **i** and **k**. Significant differences at *P* < 0.05 are indicated by letters. See Source data for exact *P* values. All experiments were repeated three times with similar results, except for **k**, which was repeated twice. Source data

The flg22^Atum^ of *A. tumefaciens* was one of the first flg22 variants reported to evade recognition by plants^3^. Previous studies on flg22^Atum^ responsiveness of FLS2 homologues in cultivated grapes (*V. vinifera*) and riverbank grapes (*V. riparia*) provide an excellent model for investigating how a novel recognition specificity is formed (Extended Data Fig. 1a)^16,24,35^. Therefore, we aimed to identify minimal key residues from the flg22^Atum^-responsive VrFLS2XL to render the flg22^Atum^-blind VvFLS2 responsive to flg22^Atum^.

**Fig. 1:**
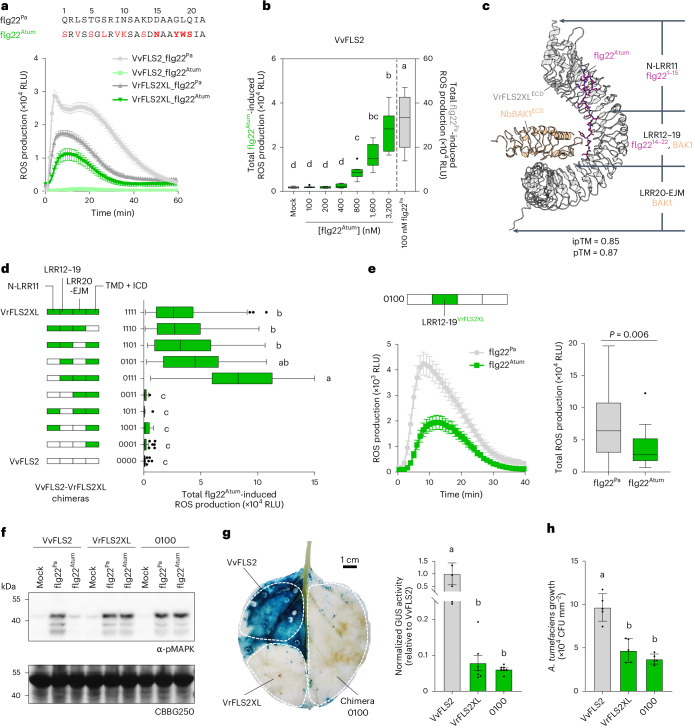
LRR12-19 is mainly responsible for flg22^Atum^ recognition by VrFLS2XL. **a**, Sequences of flg22^Atum^ and flg22^Pa^, and ROS production (in RLU) induced by VvFLS2 and VrFLS2XL in response to these peptides (*n* = 12 leaf discs). Mutations in flg22^Atum^ are highlighted in red, with reported key mutations (15N, 18Y, 19W, 20S) in boldface and shaded. **b**, Total ROS production triggered by VvFLS2 in response to flg22^Pa^ and increasing concentrations of flg22^Atum^ (*n* = 8 leaf discs). **c**, AlphaFold3-predicted structure of VrFLS2XL^ECD^-flg22^Atum^-NbBAK1^ECD^ and the segmentation scheme of VrFLS2XL for domain swapping (ECD, extracellular domain; EJM, EJM domain). NbBAK1 is also known as NbSERK3B. The ipTM and pTM values stand for the interface predicted template modelling (ipTM) score and the predicted template modelling (pTM) score. **d**, Flg22^Atum^-induced ROS production of VvFLS2-VrFLS2XL chimeras (*n* = 36, 52, 16, 28, 16, 40, 76, 48, 36, 64 leaf discs, from top to bottom). **e**, Flg22^Atum^-induced ROS production of chimera 0100 (*n* = 28 leaf discs). **f**, Phosphorylation of MAPKs by FLS2 variants in response to flg22^Pa^ and flg22^Atum^. **g**, Histochemical (left) and fluorometric (right) GUS assays showing the effects of FLS2 variants on restricting the T-DNA transfer of *A. tumefaciens* carrying a GUS reporter gene (*n* = 7 biological replicates for the fluorometric assay). White dashed lines indicate regions where FLS2s are expressed. Dark blue colouration indicates high GUS activity and a higher degree of T-DNA transfer. **h**, Significant restriction of *A. tumefaciens* growth by VrFLS2XL and 0100 compared to VvFLS2 measured by counting of colony-forming units (CFU) (*n* = 5 biological replicates). For bar plots and line charts, data are shown as mean ± s.e.m., except for **h**, where mean ± s.d. is used. For box plots: centre line, median; box limits, upper and lower quartiles; whiskers, 1.5 times interquartile range; points, outliers. Kruskal–Wallis test followed by Dunn’s multiple comparisons test was used for statistical analysis in **b**, **d**, **g** and **h**. See Source data for exact *P* values. Significant differences at *P* < 0.05 are denoted by letters. Two-tailed Mann–Whitney test was used for statistical analysis in **e**. All experiments were repeated three times with similar results, except for **h**, which was repeated twice. Source data

We first assessed the flg22^Atum^ responsiveness of VvFLS2 and VrFLS2XL in *N. benthamiana fls2* mutant (Fig. 1a,b). As a control, we concurrently tested each FLS2 variant’s response to the canonical flg22 of *Pseudomonas aeruginosa* (flg22^Pa^) to confirm that the tested FLS2 variant retains its original flg22 recognition capability. Flg22^Atum^ induced ROS production when VrFLS2XL was expressed, but not with VvFLS2 (Fig. 1a). However, VvFLS2 is not completely irresponsive to flg22^Atum^ as weak ROS production could be detected at higher flg22^Atum^ concentrations (Fig. 1b and Extended Data Fig. 1b). We also tested the flg22^Atum^ responsiveness of other FLS2 homologues, including SlFLS2 from tomato (*Solanum lycopersicum*), AtFLS2 from *Arabidopsis thaliana*, GmFLS2a/b from soybean (*G. max*) and NbFLS2 from *N. benthamiana* (Extended Data Fig. 1c–g). All these FLS2s were blind to flg22^Atum^, and flg22^Atum^ had no antagonistic effect on flg22^Pa^-induced ROS production. These results categorize flg22^Atum^ as a weak agonist^22^ that evades FLS2 recognition primarily due to the loss of binding (consistent with previous binding assays^22,24,36^). However, we cannot exclude the possibility that 18Y, 19W and 20S (hereinafter referred to as ^18^YWS^20^) mutations on flg22^Atum^ might also affect BAK1 recruitment, as suggested in previous studies^23,24^.

VvFLS2 and VrFLS2XL exhibit 82.5% protein sequence identity with more than 200 polymorphic residues. To map the minimal sequence determinants for flg22^Atum^ recognition, we first used a chimeric receptor/domain swapping strategy. According to the AlphaFold3 (ref. ^37^)-predicted structure of VrFLS2XL^ECD^-flg22^Atum^-NbBAK1^ECD^ complex (Fig. 1c) and the solved structure of AtFLS2^ECD^-flg22^Pa^-AtBAK1^ECD^ complex^6^, we divided FLS2 into four fragments: (1) N-LRR11, from the N terminus (signal peptide was included when making DNA constructs and removed when making the structural prediction) to the 11th LRR, which recognizes the first 15 amino acids of flg22; (2) LRR12-19, which interacts with amino acids 14–22 of flg22 and provides the first interacting interface for BAK1 recruitment; (3) LRR20-extracellular juxtamembrane (EJM), from LRR20 to the end of the EJM domain, which does not interact with flg22 but includes the direct FLS2–BAK1 interface; and (4) the transmembrane domain and the intracellular domain (TMD + ICD). Using Golden Gate cloning^38^, we constructed a series of chimeric receptors combining fragments of VvFLS2 and VrFLS2XL. As shown in Fig. 1d and Extended Data Fig. 1h, chimeras responded to flg22^Atum^ in a binary manner. All flg22^Atum^-responsive chimeras carry LRR12-19 of VrFLS2XL, while VrFLS2XL with its LRR12-19 replaced by LRR12-19^VvFLS2^ (chimera ‘1011’) could not respond to flg22^Atum^. Conversely, VvFLS2 carrying LRR12-19^VrFLS2XL^ (chimera ‘0100’) gained responsiveness to flg22^Atum^ (Fig. 1e). These results indicate that LRR12-19^VrFLS2XL^ is necessary for VrFLS2XL to recognize flg22^Atum^, and transplanting LRR12-19^VrFLS2XL^ is sufficient to enable VvFLS2 to recognize flg22^Atum^, while other domains might influence the recognition quantitatively.

It is worth noting that all flg22^Atum^-responsive chimeras exhibited weaker ROS responses to flg22^Pa^ than flg22^Atum^-irresponsive chimeras (Fig. 1a and Extended Data Fig. 1h,i). It is possibly because *A. tumefaciens* remaining on the infiltrated leaves is recognized by flg22^Atum^-responsive chimeric FLS2s, which can reduce the transformation rate as well as deplete signalling components and substrates for ROS production before conducting measurements. This is supported by the observation of occasional weak cell death on young leaves expressing flg22^Atum^-responsive chimeras for more than 3 days but not on leaves expressing flg22^Atum^-irresponsive chimeras. It is unlikely that the gain of flg22^Atum^ recognition per se affects flg22^Pa^ recognition, as VrFLS2XL has been previously shown to respond to flg22^Pa^ at a similar or stronger level than VrFLS2 when stably expressed in *N. benthamiana* or transiently expressed in *A. thaliana* protoplasts^24^.

In addition to ROS, we also detected flg22^Atum^-induced MAPK phosphorylation in chimera 0100, further confirming its ability to recognize flg22^Atum^ (Fig. 1f). Finally, we tested whether chimera 0100 could confer resistance to *A. tumefaciens* (Extended Data Fig. 1j). To assess the efficiency of transfer-DNA (T-DNA) transfer, we used *A. tumefaciens* GV3101 carrying a binary plasmid with an intron-containing β-glucuronidase (*GUS*) gene^39,40^ to infect *N. benthamiana* leaves transiently expressing VvFLS2, VrFLS2XL or the chimera 0100. Both histochemical and fluorometric GUS assays showed that the chimera 0100 effectively restricted T-DNA transfer to a similar extent as VrFLS2XL (Fig. 1g). As for T-DNA transfer, restriction of *A. tumefaciens* growth was also observed by measuring the bioluminescence of ‘AgroLux’ strain^41^ (Extended Data Fig. 1k) and quantifying colony-forming units (Fig. 1h). Collectively, our results show that the LRR12-19 region of VrFLS2XL is primarily responsible for flg22^Atum^ recognition.

### VrFLS2XL targets key evading mutations to recognize flg22^Atum^

**Fig. 2:**
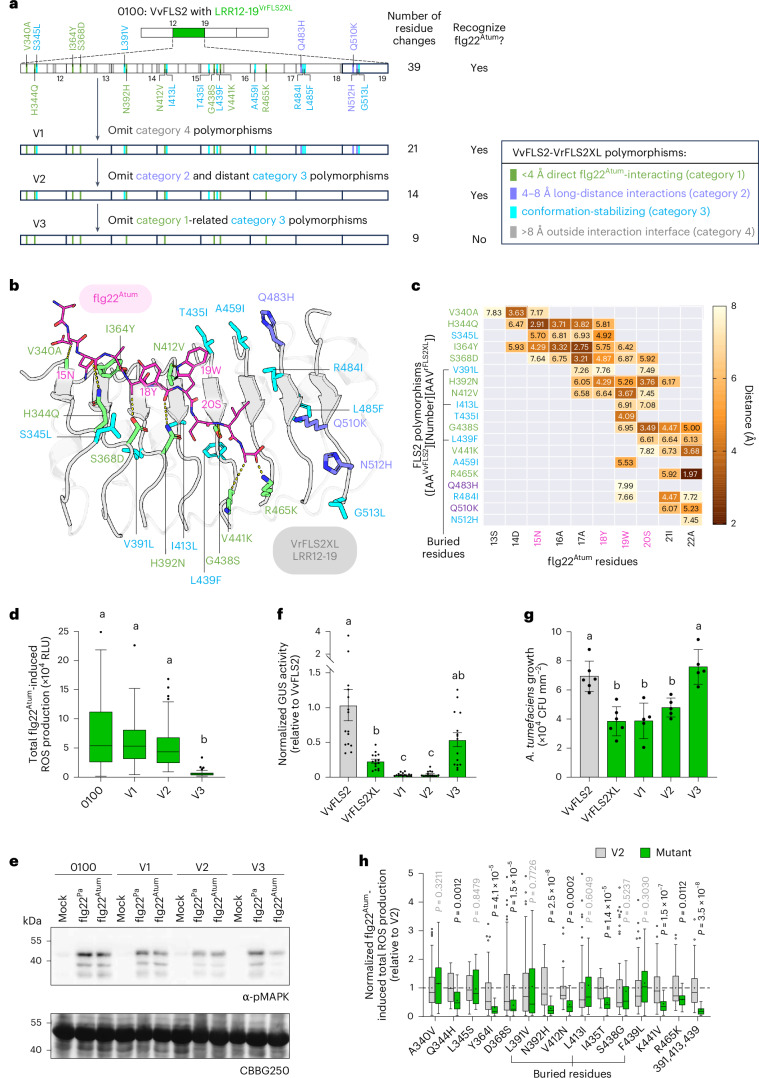
Computer-aided identification and characterization of minimal key residues of VrFLS2XL responsible for flg22^Atum^ recognition. **a**, Schematic overview of polymorphic sites between LRR12-19^VrFLS2XL^ and VvFLS2, and the process to identify minimal key residues. To label a polymorphic site, the first and the second letter indicate residue status in VvFLS2 and VrFLS2XL, respectively. The number indicates the residue position in VrFLS2XL. Panels **a**–**c** share labels and legends. **b**, AlphaFold3-predicted interaction interface of LRR12-19^VrFLS2XL^ and flg22^Atum^. Potential flg22^Atum^-interacting polymorphic residues are displayed with side chains and colour-coded. **c**, Contact map showing all interaction pairs with distances <8 Å. Numbers indicate the distances. AA, amino acid. **d**, Comparison of total flg22^Atum^-induced ROS production of VvFLS2 variants listed in **a**, *n* = 61, 60, 80, 39 leaf discs, from left to right. **e**, Phosphorylation of MAPKs by FLS2 variants in response to flg22^Pa^ and flg22^Atum^. **f**, Restriction of *A. tumefaciens* T-DNA transfer by natural and engineered FLS2 variants (*n* = 15 biological replicates). **g**, Restriction of *A. tumefaciens* growth by natural and engineered FLS2 variants (*n* = 6, 6, 5, 5, 5 biological replicates). **h**, Characterization of individual residues in V2. Total flg22^Atum^-induced ROS production was normalized by the average value of V2. ‘391,413,439’ denotes three mutations L391V, L413I and F439L at buried sites. The dotted line indicates *Y* = 1. For V2, *n* = 68, 24, 24, 76, 100, 96, 44, 28, 72, 24, 68, 56, 68, 36, 40 leaf discs, from left to right. For mutants, *n* = 40, 24, 24, 76, 68, 96, 44, 28, 72, 24, 68, 56, 68, 24, 40 leaf discs, from left to right. For bar plots, data are shown as mean ± s.e.m., except for **g**, where mean ± s.d. is used. Kruskal–Wallis test followed by Dunn’s multiple comparisons test was used for statistical analysis in **d**, **f** and **g**. Significant differences at *P* < 0.05 are denoted by letters. Two-tailed Mann–Whitney test was used for statistical analysis in **h**. See Methods section and Source data for more information about statistical analyses. All experiments were repeated three times with similar results, except for **g**, which was repeated twice. Source data

**Extended Data Fig. 2:**
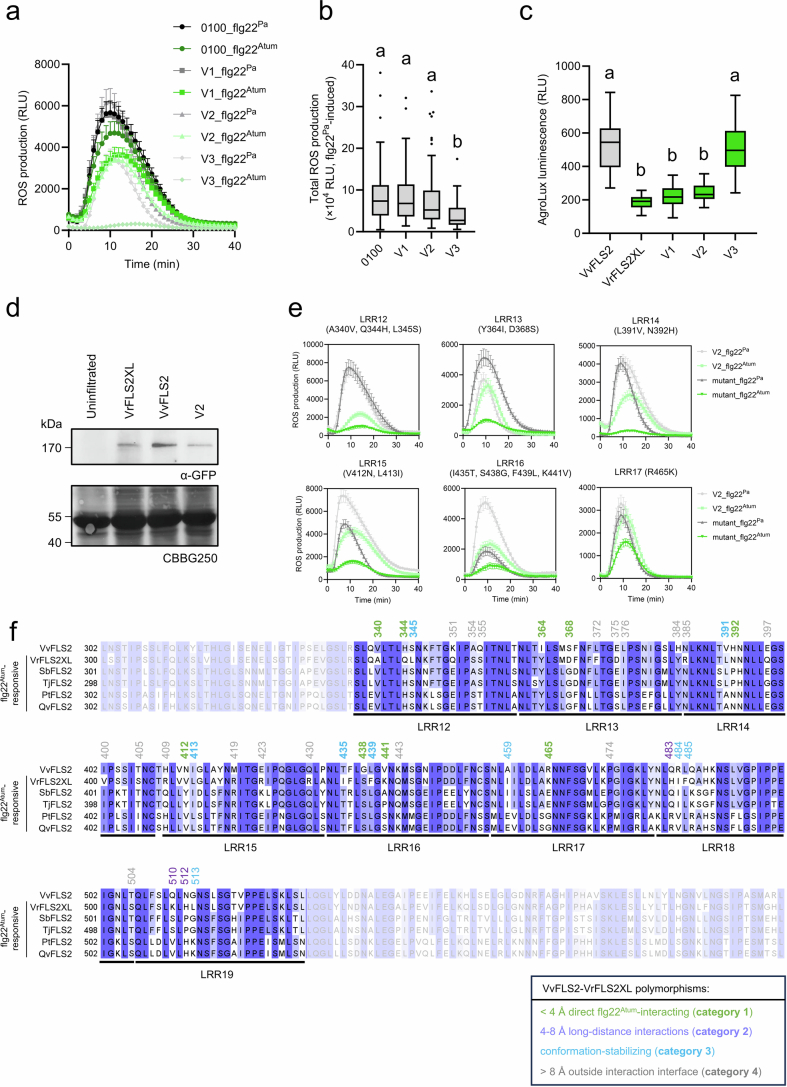
Characterization of engineered VvFLS2 variants. **a**, Flg22^Pa^- and flg22^Atum^-induced ROS production of engineered FLS2 variants listed in Fig. 2a (corresponding to Fig. 2d, *n* = 64, 61, 56, 60, 84, 80, 40, 39 leaf discs, from top to bottom). **b**, Comparison of total flg22^Pa^-induced ROS production among engineered VvFLS2 variants (*n* = 64, 56, 84, 40 leaf discs). **c**, Restriction of *A. tumefaciens* growth by engineered VvFLS2 variants (*n* = 48 leaf discs), with bioluminescence of the ‘AgroLux’ strain used as a proxy for bacterial population size. **d**, Protein expression levels of GFP-tagged VvFLS2, VrFLS2XL, and ‘V2’. **e**, Effects of mutating clusters of polymorphic residues in each LRR from LRR12-17 of ‘V2’ (*n* = 24, 24, 20, 48, 12, 36 leaf discs from group ‘LRR12’ to group ‘LRR17’). **f**, Sequence alignment of the LRR12-19 region of VvFLS2, VrFLS2XL, and other previously reported flg22^Atum^-responsive FLS2 variants. Polymorphic sites between VvFLS2 and VrFLS2XL are marked with their position within VrFLS2XL. Polymorphic residue numbers in ‘V2’ variant are shown in bold. The legends are consistent with Fig. 2a. LRR annotations are based on ‘4MN8’ structure from the Protein Data Bank (PDB). (Qv, *Quercus variabilis*; Tj, *Trachelospermum jasminoides*; Sb, *Salix babylonica*; Pt, *Populus trichocarpa*). For line charts, data are presented as mean ± SEM. For box plots: center line, median; box limits, upper and lower quartiles; whiskers, 1.5 times interquartile range; points, outliers. Kruskal-Wallis test followed by Dunn’s multiple comparisons test was used for statistical analysis in **b** and **c**. Significant differences at *P* < 0.05 are indicated by letters. See Source data for exact *P* values. Sequence alignment and visualization were performed using Jalview^69^. All experiments were repeated three times with similar results, except for **c**, which was repeated twice. Source data

Between VvFLS2 and VrFLS2XL, there are a total of 39 polymorphic sites within the LRR12-19 region (Fig. 2a and Extended Data Fig. 2f). To identify the minimal key residues for flg22^Atum^ recognition, we first evaluated the roles of polymorphic sites based on the AlphaFold3-predicted structure (Fig. 1c and Fig. 2b). Given that no polymorphic site was predicted to directly interact with BAK1, we grouped the 39 polymorphic sites into four categories (see Fig. 2a legends) based on their predicted distance from flg22^Atum^ (Fig. 2c): (1) residues directly interacting with flg22^Atum^ at a distance less than 4 Å, (2) residues putatively in contact with flg22^Atum^ potentially through long-distance (4–8 Å) electrostatic interactions and water-mediated contacts^42^, (3) neighbouring residues of categories 1 and 2 residues potentially stabilizing the local conformation (although these neighbouring residues may not directly contribute to binding interactions, they appear to support the ligand binding by stabilizing the geometric conformation of local motifs, such as the distance between repeat modules and protein curvatures^43,44^) and (4) residues outside the predicted interaction interface (distance >8 Å).

We then refined the polymorphic sites in a stepwise manner by mutating VrFLS2XL residues to VvFLS2 residues (Fig. 2a and Extended Data Fig. 2f). First, we omitted 18 polymorphic residues outside the interaction interface (category 4; see Extended Data Fig. 2f for exact residue numbers), generating a variant called ‘V1’. V1 exhibited only a slight and insignificant reduction in flg22^Atum^ and flg22^Pa^ recognition capabilities compared to chimera 0100 (Fig. 2d and Extended Data Fig. 2a,b). Then, we created ‘V2’ by omitting category 2 residues (Q483H, Q510K, N512H) and distant category 3 residues (A459I, R484I, L485F, G513L) from V1. This step again resulted in a minimal decrease in both flg22^Pa^ and flg22^Atum^ recognition capabilities (Fig. 2d and Extended Data Fig. 2a,b). However, when we further omitted neighbouring polymorphic residues of category 1 residues (‘category 1-related category 3 residues’ in Fig. 2a: S345L, V391L, I413L, T435I, L439F) to generate ‘V3’, we observed a significant reduction in both flg22^Pa^ and flg22^Atum^ recognition capabilities (Fig. 2d and Extended Data Fig. 2a,b). In line with the ROS results, V3 also exhibited compromised flg22^Atum^-induced MAPK phosphorylation compared to 0100, V1 and V2 (Fig. 2e). In terms of conferring resistance to *A. tumefaciens*, V1 and V2 effectively restricted T-DNA transfer and bacterial growth to a level comparable and even superior to VrFLS2XL, while V3 exhibited no significant difference from VvFLS2 (Fig. 2f,g and Extended Data Fig. 2c). The gain of flg22^Atum^ recognition by V2 was not caused by an increased protein accumulation level (Extended Data Fig. 2d).

**Extended Data Fig. 3:**
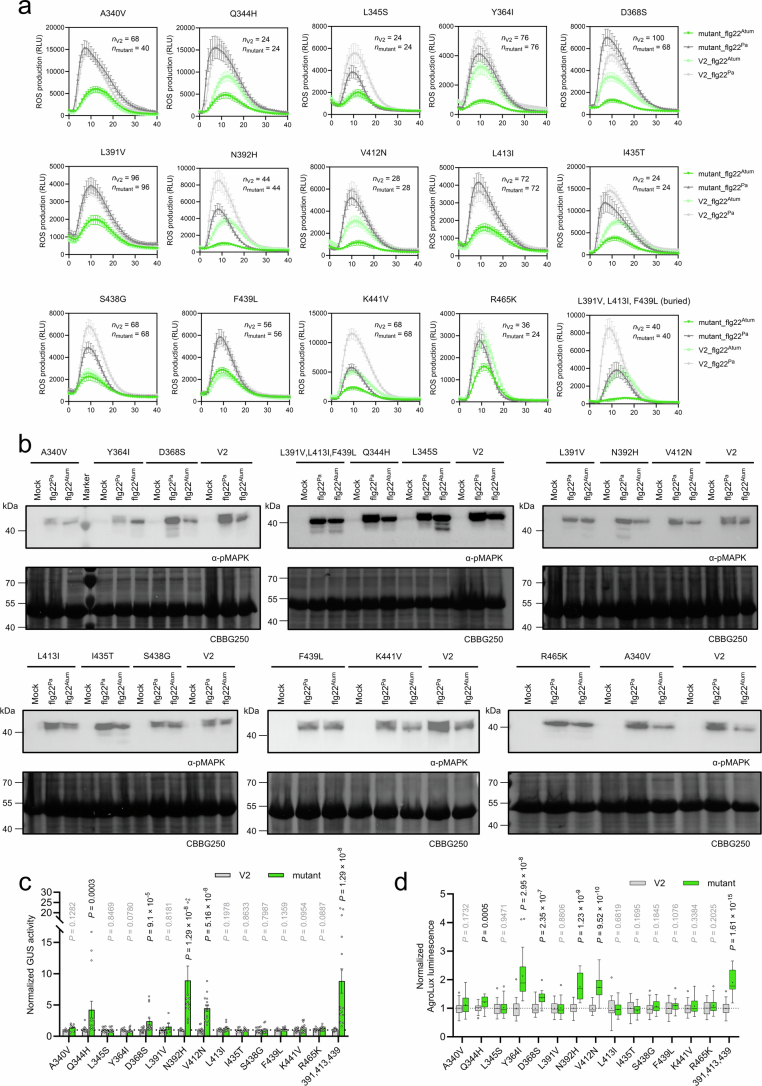
Characterization of polymorphic residues in the engineered variant ‘V2’. Flg22^Pa^- and flg22^Atum^-induced ROS burst kinetics (**a**) and MAPK phosphorylation (**b**), inhibition of *A. tumefaciens*-mediated T-DNA transfer (**c**), and suppression of *A. tumefaciens* growth (**d**) by engineered variant ‘V2’ carrying single or multiple mutations at polymorphic sites (related to Fig. 2h). Number of leaf discs measured in **a** are indicated in the plots (*n*_V2_ and *n*_mutant_). For each FLS2 variant, flg22^Pa^ and flg22^Atum^ treatment groups have the same sample sizes. Samples sizes of pairs in **c**: *n* = 7, 15, 11, 12, 16, 6, 15, 15, 12, 9, 12, 9, 15, 12, 15 biological replicates, from left to right. Samples sizes of pairs in **d**: *n* = 32 leaf discs for ‘391,413,419’; *n* = 28 leaf discs for ‘S438G’, ‘F439L’, ‘K441V’; *n* = 24 leaf discs for the rest. For line charts and bar plots, data are shown as mean ± SEM. For box plots: center line, median; box limits, upper and lower quartiles; whiskers, 1.5 times interquartile range; points, outliers; ‘+’, mean. The dotted line indicates Y = 1. Two-tailed unpaired t test was used for mutants ‘A340V’, ‘D368S’, ‘L391V’, ‘V412N’, ‘L413I’, ‘I435T’, ‘F439L’, and ‘R465K’ in **d**. Two-tailed Mann–Whitney test was used for statistical analysis in **c**, and for mutants ‘Q344H’, ‘L345S’, ‘Y364I’, ‘N392H’, ‘S438G’, ‘K441V’, and ‘391,413,419’ in **d**. Similar results were obtained from two (**b**, **c**, **d**) or three (**a**) independent experiments. Source data

To further refine V2, we first evaluated each LRR carrying polymorphic residues (Extended Data Fig. 2e). Given that every LRR of LRR12-17 seemed to be indispensable for flg22^Atum^ recognition, we then individually characterized each polymorphic residue of V2 (Fig. 2h and Extended Data Fig. 3a). Most solvent-exposed residues are important for flg22^Atum^ recognition, except 340A and 345L (Fig. 2h). N392H and K441V mutations affect not only flg22^Atum^ recognition but also flg22^Pa^ recognition (Extended Data Fig. 3a). For buried residues (391L, 413L and 439F), while single mutation did not affect flg22^Atum^ recognition, mutating all of them abolished flg22^Atum^ recognition and compromised flg22^Pa^ recognition (Fig. 2h and Extended Data Fig. 3a).

In addition to ROS production, we also evaluated the effects of these mutations on MAPK phosphorylation and *A. tumefaciens* resistance. However, the MAPK phosphorylation assay lacked the sensitivity and quantitative resolution to detect these changes reliably (Extended Data Fig. 3b). It is worth noting that among all mutants exhibiting reduced flg22^Atum^-induced ROS production, only Q344H, D368S, N392H, V412N and the triple buried residue mutant L391V/L413I/F439L consistently showed impaired ability to restrict both T-DNA transfer and bacterial growth (Extended Data Fig. 3c,d), highlighting the critical roles of these residues. These findings also suggest that surpassing a threshold level of flg22^Atum^ recognition capability is sufficient to confer effective resistance against *A. tumefaciens* and that further enhancing recognition beyond this threshold does not lead to additional resistance.

It is worth noting that several key residues found in VrFLS2XL are also present in other reported flg22^Atum^-responsive FLS2 variants^26^ (Extended Data Fig. 2f). For example, 364Y and 438S are found in all five reported flg22^Atum^-responsive FLS2 variants^26^, while 392N and 412V appear in three of them, implying that plants might have independently evolved similar mechanisms to recognize flg22^Atum^.

It is worth noting that all polymorphic residues critical for flg22^Atum^ recognition are predicted to be located within the flg22^Atum^-interacting interface (Fig. 2b,c). Most of them are predicted to directly interact with key evading mutations of flg22^Atum^ (15N, ^18^YWS^20^) or adjacent residues. The exceptions are 441K and 465K, which are predicted to interact with the C-terminus of flg22^Atum^, likely through electrostatic interactions, which may further strengthen the binding of flg22 to FLS2. These findings suggest that optimizing FLS2–flg22 interaction around flg22’s key evasion sites could be a viable strategy for engineering recognition towards weak agonistic flg22 variants. Optimization of FLS2–flg22 interaction can be achieved by either increasing the binding affinity or accommodating flg22 in a manner to better interact with BAK1^24^.

### BAK1-interacting LRR20-EJM^GmFLS2b^ mediates flg22^Rso^ recognition

**Fig. 3:**
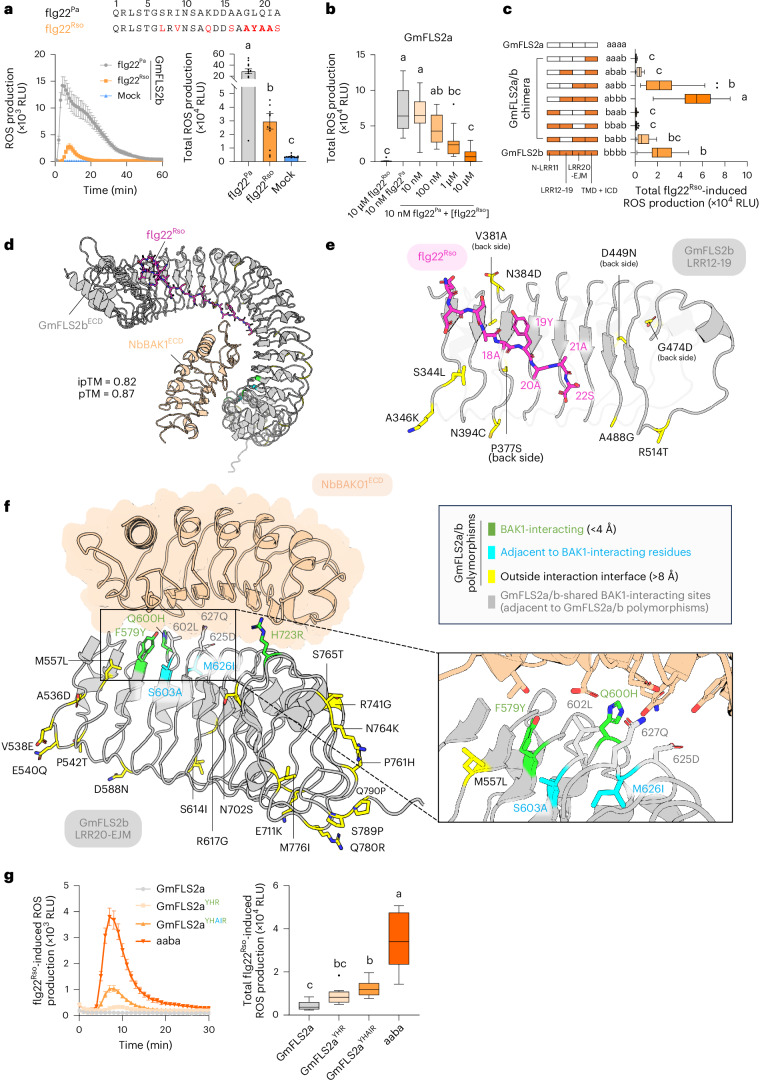
Domain swapping and structural modelling reveal critical polymorphic residues on the direct FLS2–BAK1 interaction interface of LRR20-EJM^GmFLS2b^ for antagonistic flg22^Rso^ recognition. **a**, Sequences of flg22^Rso^ and flg22^Pa^, and GmFLS2b’s ROS responses to them (*n* = 12 leaf discs). Mutations in flg22^Rso^ are marked in red. Reported key evading mutations are in bold and shaded. **b**, ROS responses of GmFLS2a induced by flg22^Rso^ and flg22^Pa^ with increasing concentrations of flg22^Rso^ (*n* = 16 leaf discs). **c**, Flg22^Rso^-induced ROS responses of GmFLS2a/b chimeras (*n* = 22, 32, 57, 40, 19, 24, 12, 44 leaf discs, from top to bottom). **d**, Overview of the AlphaFold3 (AF3)-predicted GmFLS2b^ECD^-flg22^Rso^-NbBAK1^ECD^ complex structure. ECD, extracellular domain. **e**, AF3-predicted structure of GmFLS2b^LRR12-19^ in complex with C-terminal flg22^Rso^. All polymorphic sites lie outside the interaction interface with flg22 and BAK1. **f**, AF3-predicted structure of GmFLS2b^LRR20-EJM^-NbBAK1^ECD^ complex. The left panel provides an overview; the right panel zooms in on the direct FLS2–BAK1 interaction interface. Polymorphic residues are displayed with side chains and colour-coded in green, cyan and yellow based on categories from the legends. Three direct BAK1-interacting residues with no difference between GmFLS2a/b are also shown because they are adjacent to polymorphic sites. **g**, Flg22^Rso^-induced ROS responses of GmFLS2a carrying predicted critical residues, compared to GmFLS2a and chimera aaba (*n* = 12 leaf discs). Total ROS production over 30 min was calculated. YHR, (F579Y, Q600H, H723R); YHAIR, (F579Y, Q600H, S603A, M626I, H723R). For bar plots and line charts, data are shown as mean ± s.e.m. For box plots: centre line, median; box limits, upper and lower quartiles; whiskers, 1.5 times interquartile range; points, outliers; ‘+’, mean. Kruskal–Wallis test followed by Dunn’s multiple comparisons test was used for statistical analysis in **a**–**c**. One-way ANOVA with Tukey’s test was used for statistical analysis in **g**. Significant differences at *P* < 0.05 are denoted by letters. See Source data for exact *P* values. All experiments were repeated at least three times with similar results. Source data

Instead of losing binding to FLS2, some flg22 variants retain the ability to interact with FLS2 but impede BAK1 recruitment, acting as antagonists of FLS2-induced signalling. A notable example is flg22^Rso^, which was reported to be a weak antagonist for AtFLS2 and a strong antagonist for SlFLS2^21,33^. The ^18^AYA^20^ motif and 21A mutations have been identified as critical for this antagonism (Fig. 3a)^25,33^.

**Extended Data Fig. 4:**
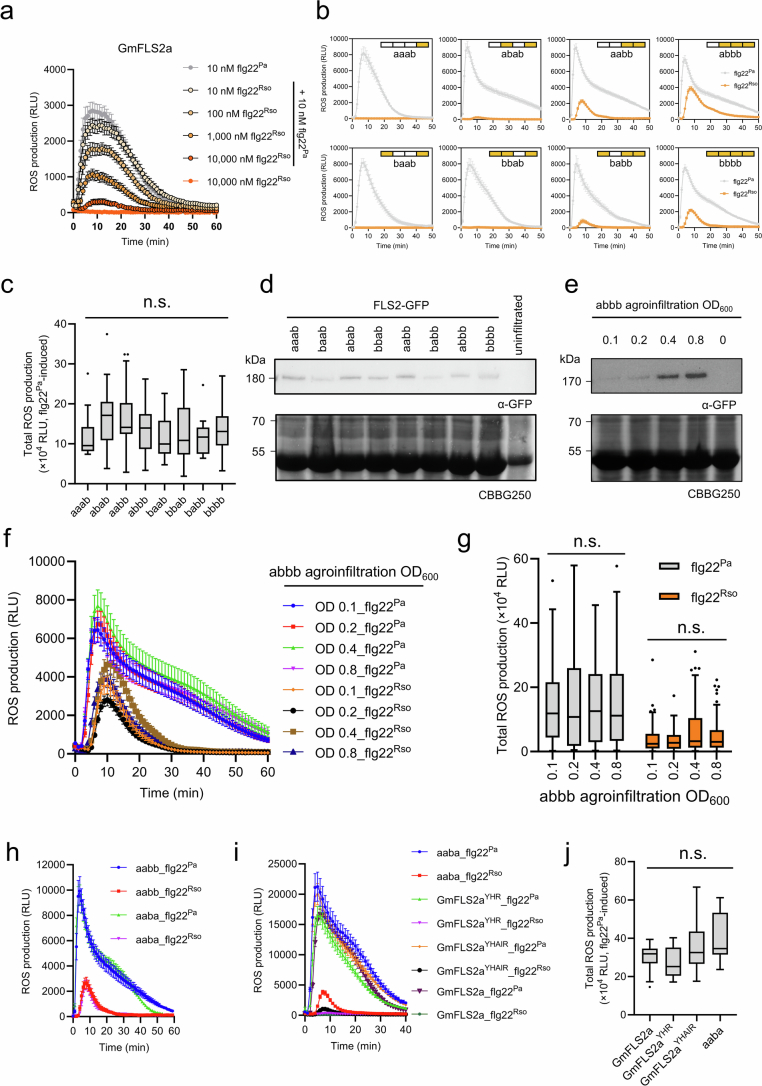
ROS burst kinetics and total flg22^Pa^-induced ROS production of GmFLS2a/b chimeras and GmFLS2a variants with predicted gain-of-recognition polymorphic residues. **a**, ROS burst kinetics corresponding to Fig. 3b. **b**–**d**, ROS burst kinetics (**b**), total flg22^Pa^-induced ROS production (**c**), and protein expression levels (**d**) of GmFLS2a/b chimeras (related to Fig. 3c). **e**, Protein expression levels of chimera ‘abbb’ at varying agroinfiltration OD_600_ values. **f**, **g**, Flg22^Pa^- and flg22^Rso^-induced ROS production kinetics (**f**) and total ROS production (**g**) of chimera ‘abbb’ at varying agroinfiltration OD_600_ values (*n* = 72 leaf discs). **h**, Flg22^Pa^- and flg22^Rso^-induced ROS responses of ‘aabb’ and ‘aaba’ chimeras (*n* = 12 leaf discs). **i**, **j**, ROS production kinetics (**i**), and total flg22^Pa^-induced ROS production (**j**) of GmFLS2a variants carrying predicted gain-of-recognition residues (related to Fig. 3g, *n* = 12 leaf discs). For line charts, data are presented as mean ± SEM. For box plots: center line, median; box limits, upper and lower quartiles; whiskers, 1.5 times interquartile range; points, outliers. Kruskal-Wallis test followed by Dunn’s multiple comparisons test was used for statistical analysis of **c**, **g**, and **j**. ‘n.s.’ indicates no significant difference. See Source data for exact *P* values. Similar results were obtained from two (**d**–**g**) or three (**a**–**c**, **h**–**j**) independent experiments. Source data

GmFLS2b was the first FLS2 variant reported to recognize flg22^Rso^ (ref. ^25^). By contrast, GmFLS2a, another copy of soybean FLS2 with 90.6% protein sequence identity to GmFLS2b, does not respond to flg22^Rso^ when expressed alone in *N. benthamiana*. Before studying them, we first validated the flg22^Rso^ responsiveness of GmFLS2a and GmFLS2b using the *N. benthamiana fls2* mutant. GmFLS2b could recognize flg22^Rso^, but the ROS production induced by flg22^Rso^ was significantly weaker than that induced by flg22^Pa^ (Fig. 3a). GmFLS2a, however, was irresponsive to flg22^Rso^, even at a high concentration (10 μM), and flg22^Rso^ acted as an antagonist of flg22^Pa^-induced ROS production for GmFLS2a (Fig. 3b and Extended Data Fig. 4a).

To map the minimal sequence determinants required for flg22^Rso^ recognition by GmFLS2b, we first performed domain swapping between GmFLS2a and GmFLS2b, using the same segmentation scheme as in the VrFLS2XL case. The resulting chimeras exhibited varying levels of flg22^Rso^ recognition (Fig. 3c and Extended Data Fig. 4b), although they responded similarly to flg22^Pa^ (Extended Data Fig. 4b,c). It is worth noting that the chimera ‘aabb’, which contains the LRR20-EJM domain of GmFLS2b, responded to flg22^Rso^ at a level comparable to GmFLS2b, indicating that LRR20-EJM^GmFLS2b^ is mainly responsible for flg22^Rso^ recognition. While LRR12-19^GmFLS2b^ only rendered a slight and statistically insignificant ROS response to flg22^Rso^ (chimera ‘abab’), its combination with LRR20-EJM^GmFLS2b^ (chimera ‘abbb’) significantly enhanced flg22^Rso^ recognition. It is worth noting that N-LRR11^GmFLS2b^ negatively influenced flg22^Rso^ perception, as chimeras lacking N-LRR11^GmFLS2b^ exhibited a stronger flg22^Rso^-induced ROS production than those containing it. Despite the lower protein expression levels of chimeras carrying N-LRR11^GmFLS2b^ compared to their counterparts with N-LRR11^GmFLS2a^ (Extended Data Fig. 4d), the negative effect of N-LRR11^GmFLS2b^ on flg22^Rso^ perception is unlikely due to the reduced protein expression levels. This conclusion is supported by the observation that varying expression levels of chimera abbb, achieved using a gradient of agroinfiltration OD_600_ values (Extended Data Fig. 4e), exhibited similar flg22^Pa^- and flg22^Rso^-induced ROS production levels (Extended Data Fig. 4f,g). Therefore, we speculate that N-LRR11^GmFLS2b^ interacts less effectively with flg22^Rso^ than N-LRR11^GmFLS2a^. The TMD and ICD did not appear to affect flg22^Rso^ perception (Extended Data Fig. 4h).

As the LRR20-EJM region interacts exclusively with BAK1 and antagonistic flg22^Rso^ impedes BAK1 recruitment, we hypothesized that specific BAK1-interacting residues within LRR20-EJM^GmFLS2b^ could strengthen the FLS2–BAK1 interaction to overcome flg22^Rso^ antagonism. Based on the AlphaFold3-predicted structure (Fig. 3d,f), three polymorphic residues—F579Y, Q600H and H723R—directly interact with NbBAK1. However, these changes were insufficient to generate statistically significant ROS production in response to flg22^Rso^ (GmFLS2a^YHR^ in Fig. 3g). Building on insights from the VrFLS2XL case, we introduced two additional mutations, S603A and M626I, adjacent to the direct interaction sites shared by GmFLS2a/b (602L, 627Q and 625D). This resulted in weak but statistically significant ROS production in response to flg22^Rso^ (GmFLS2a^YHAIR^ in Fig. 3g). GmFLS2a^YHR^ and GmFLS2a^YHAIR^ responded to flg22^Pa^ similar to GmFLS2a and chimera ‘aaba’ (Extended Data Fig. 4i,j).

Given LRR12-19^GmFLS2b^’s significant contribution to flg22^Rso^ recognition, we also examined the predicted structure of LRR12-19^GmFLS2b^ (Fig. 3e). Surprisingly, all polymorphic sites within LRR12-19^GmFLS2b^ are predicted to be outside the flg22- or BAK1-interacting interface, suggesting that these residues may influence recognition specificity through unknown mechanisms, for example, modulating interactions with other regulators.

### GmFLS2b recognizes flg22^Rso^ via enhanced FLS2–BAK1 interaction

**Fig. 4:**
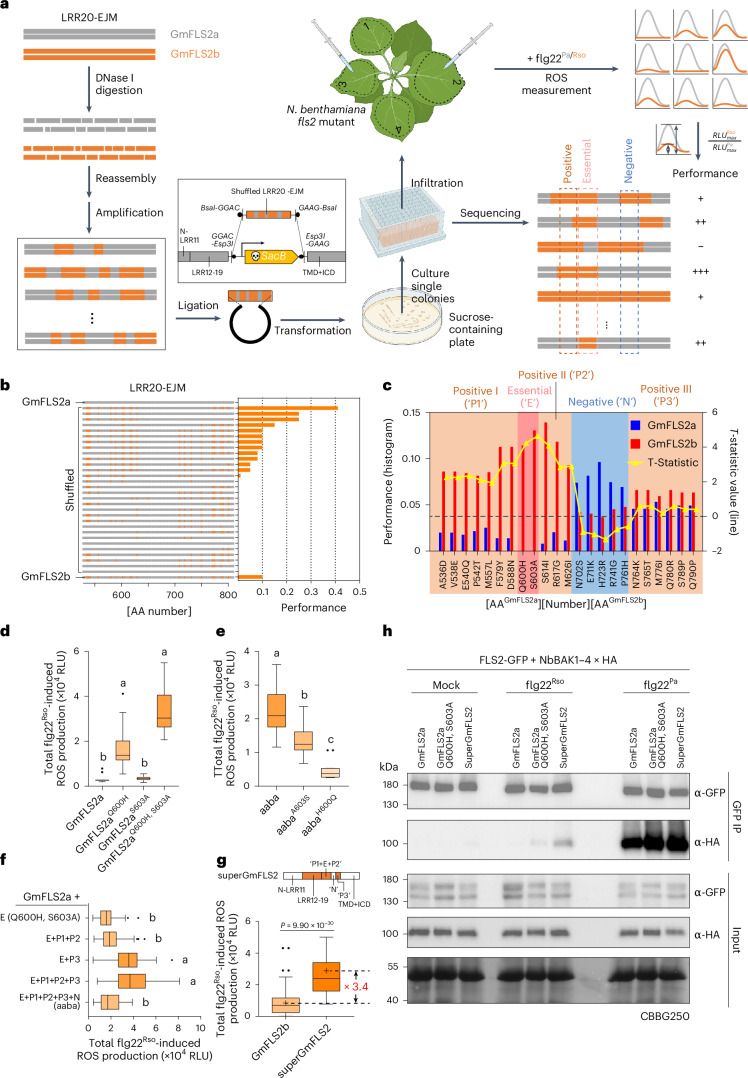
DNA shuffling dissects the roles of polymorphisms within the LRR20-EJM region of GmFLS2a/b and generates greater recognition capacity towards flg22^Rso^. **a**, Schematic diagram of the DNA shuffling workflow. See Methods section for detailed explanations. For illustrative purposes only, the numbers on the leaves indicate the serial numbers of the shuffled variants tested, while the dashed lines mark the infiltrated regions. **b**, Summary of the sequences and performances of 30 shuffled variants. **c**, Analysis of DNA shuffling results. See the Methods section for detailed explanations of this plot. **d**, Flg22^Rso^-induced ROS production of GmFLS2a with single and double mutations of H600Q and S603A (*n* = 12 leaf discs). **e**, Effects of A603S and H600Q on the flg22^Rso^-induced ROS response of chimera aaba (*n* = 12 leaf discs). **f**, Effects of DNA shuffling-predicted enhancer and ‘suppressor’ polymorphisms on flg22^Rso^-induced ROS production (*n* = 36, 80, 36, 44, 36 leaf discs, from top to bottom). See **c** for the definitions of E, P1, P2, P3 and N. **g**, Comparison of total flg22^Rso^-induced ROS production between GmFLS2b and SuperGmFLS2 (*n* = 138 and 130 leaf discs, from left to right). SuperGmFLS2 combines all the GmFLS2b-originated (orange) regions/polymorphic residues with positive effects on flg22^Rso^ recognition. **h**, Co-IP of NbBAK1 with wild-type GmFLS2a and two engineered variants. For bar plots and line charts, data are presented as mean. For box plots: centre line, median; box limits, upper and lower quartiles; whiskers, 1.5 times interquartile range; points, outliers; ‘+’, mean. Kruskal–Wallis test followed by Dunn’s multiple comparisons test was used for statistical analysis in **d**–**f** with significant differences at *P* < 0.05 indicated by letters. See Source data for exact *P* values. Two-tailed Mann–Whitney test was used for **g**. ROS experiments in **d**–**g** were repeated three times with similar results. Co-IP experiment in **h** was repeated twice with similar results. Panel **a** created with BioRender.com. Source data

The observation that GmFLS2a^YHAIR^ did not respond to flg22^Rso^ as strongly as the chimera aaba (Fig. 3g) suggests the presence of additional polymorphic residues contributing to flg22^Rso^ recognition. To identify such residues and further narrow down the minimal gain-of-recognition residues, we used DNA shuffling^45^, a method that has been successfully used to study plant immune receptors like Cf-4, Cf-9 and Pto^46,47^. As shown in Fig. 4a, DNA shuffling could generate numerous variants with different combinations of polymorphic residues from LRR20-EJM^GmFLS2a/b^. By characterizing these variants, we would be able to determine the contribution of each polymorphic residue to flg22^Rso^ recognition. To better use it in our case, we optimized the classic DNA shuffling method in two ways. First, we made it compatible with Golden Gate cloning, allowing seamless integration with the chimeric receptor construction strategy. Second, we used the broad-host-range negative selection marker *SacB* (ref. ^48^) to enable direct library construction in *A. tumefaciens* and meanwhile reduced the occurrence of false-positive clones in the resulting library.

**Extended Data Fig. 5:**
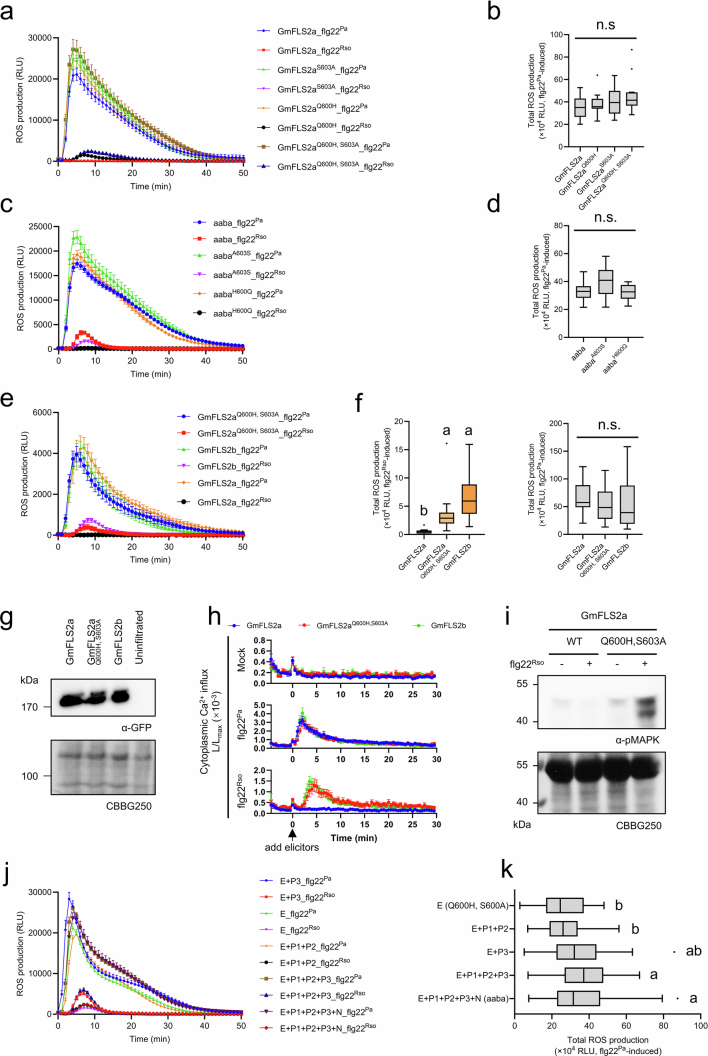
Characterization of minimal gain-of-function residues, along with ‘enhancer’ and ‘suppressor’ polymorphisms predicted by DNA shuffling. **a**, **b**, ROS production kinetics (**a**) and total flg22^Pa^-induced ROS production (**b**) of GmFLS2a and mutants (related to Fig. 4d, *n* = 12 leaf discs). **c**, **d**, ROS production kinetics (**c**) and total flg22^Pa^-induced ROS production (**d**) of chimera ‘aaba’ and mutants (related to Fig. 4e, *n* = 12 leaf discs). **e**–**i**, Flg22^Pa^- and flg22^Rso^-induced ROS burst kinetics (**e**, *n* = 16 leaf discs) and total ROS production (**f**), protein expression levels (**g**), flg22^Pa^- and flg22^Rso^-induced cytoplasmic calcium influx (**h**, *n* = 8 leaf discs), and flg22^Rso^-induced MAPK phosphorylation (**i**) of GmFLS2a carrying Q600H and S603A mutations compared to wild-type GmFLS2a and GmFLS2b. **j**, **k**, ROS burst kinetics (**j**, *n* = 36, 36, 36, 36, 84, 80, 52, 44, 36, 36 leaf discs, from top to bottom) and total flg22^Pa^-induced ROS production (**k**, *n* = 36, 36, 36, 36, 84, 80, 52, 44, 36, 36 leaf discs, from top to bottom) of engineered GmFLS2a variants carrying clusters of predicted ‘enhancer’ and ‘suppressor’ mutations (related to Fig. 4f). For line charts, data are presented as mean ± SEM. For box plots: center line, median; box limits, upper and lower quartiles; whiskers, 1.5 times interquartile range; points, outliers. Kruskal-Wallis test followed by Dunn’s multiple comparisons test was performed for **b**, **d**, **f**, and **k**, and significant differences at *P* < 0.05 are indicated by letters. See Source data for exact *P* values. The ‘n.s.’ denotes no significant difference. All experiments were repeated three times with similar results, except for **i**, which was repeated twice. Source data

Based on the characterization of 30 variants generated by DNA shuffling (Fig. 4b and Supplementary Table 3), we analysed how each polymorphic site contributes to flg22^Rso^ recognition (Fig. 4c). The results of DNA shuffling indicate that 600H and 603A of GmFLS2b are indispensable for flg22^Rso^ recognition. To assess the sufficiency and necessity of these two residues, we introduced Q600H and S603A mutations individually or together to GmFLS2a (Fig. 4d and Extended Data Fig. 5a). S603A alone did not enable GmFLS2a to respond to flg22^Rso^. While Q600H could sometimes confer a weak response to flg22^Rso^, it was not consistently statistically significant across replicates or in different contexts (for example, GmFLS2a^YHR^ in Fig. 3g). However, the combination of Q600H and S603A generated stable ROS production in response to flg22^Rso^, which was not significantly weaker than that of GmFLS2b (Extended Data Fig. 5e,f). The gain of flg22^Rso^ recognition was not due to a change in FLS2 abundance (Extended Data Fig. 5g) and was not accompanied by an increase in flg22^Pa^ responsiveness (Extended Data Fig. 5b,f). We further validated these findings by introducing H600Q and A603S mutations into the chimera aaba. H600Q completely abolished flg22-induced ROS production, while A603S significantly reduced it (Fig. 4e and Extended Data Fig. 5c,d). It has been previously reported that some flg22 variants can uncouple immune outputs, for example, triggering only a reduced ROS response but no other immune responses^21^. Therefore, we assessed the calcium influx and MAPK phosphorylation of GmFLS2a^H600Q, S603A^. Both immune outputs can be induced by flg22^Rso^ (Extended Data Fig. 5h,i). Collectively, these results show that H600Q and S603A are bona fide minimal gain-of-function mutations for GmFLS2a to recognize flg22^Rso^.

**Extended Data Fig. 6:**
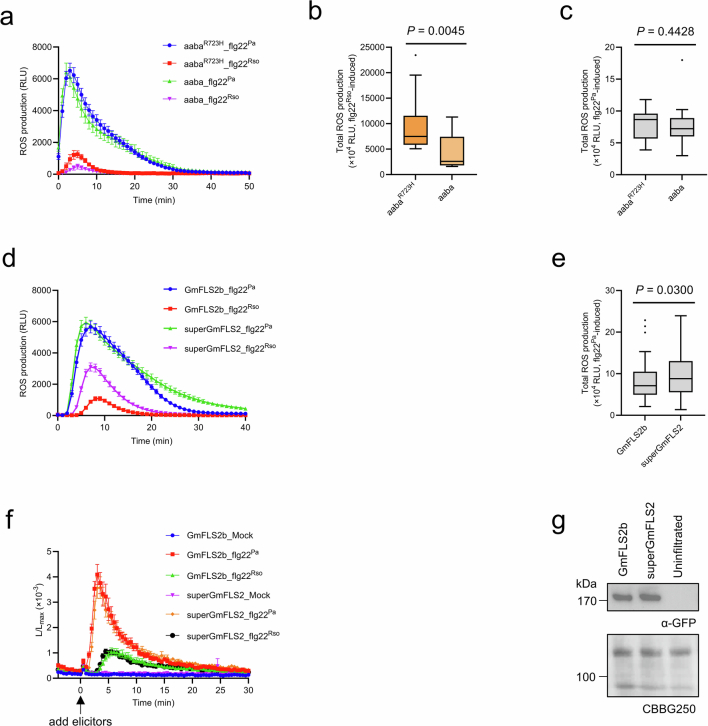
Characterization of the negative effect of R723H on flg22^Rso^ recognition and the enhanced recognition capability of the ‘superGmFLS2’ variant. **a**–**c**, Effects of the R723H mutation on flg22^Pa^- and flg22^Rso^-induced ROS production (*n* = 12 leaf discs). **d**–**g**, Comparison of ROS burst kinetics (**d**), total flg22^Pa^-induced ROS production (**e**), flg22^Pa^- and flg22^Rso^-induced cytoplasmic calcium influx (**f**, *n* = 16 leaf discs), and protein expression levels (**g**) between ‘superGmFLS2’ and GmFLS2b (related to Fig. 4g). For line charts, data are presented as mean ± SEM. For box plots: center line, median; box limits, upper and lower quartiles; whiskers, 1.5 times interquartile range; points, outliers. Two-tailed Mann-Whitney test was performed for **b**, **c**, and **e**. Similar results were obtained from three independent experiments. Source data

In addition to the two essential residues, the DNA shuffling results suggest that other polymorphic residues function as suppressors or enhancers to affect flg22^Rso^ recognition. According to their predicted functions, we categorized them into the following groups: positive I (‘P1’, from A536D to D588N), positive II (‘P2’, from S614I to M626I), negative (‘N’, from N702S to P761H) and positive III (‘P3’, from N764K to Q790P) (Fig. 4c). To verify the DNA shuffling-predicted effects of each group, we sequentially introduced them into GmFLS2a^Q600H, S603A^ (GmFLS2a with group ‘E’) (Fig. 4f and Extended Data Fig. 5j,k). The introduction of P1 and P2 resulted in a slight, but not significant, enhancement of ROS production in response to flg22^Rso^, while P3 had a significant positive effect. P3 also significantly enhanced flg22^Pa^ responsiveness, although not as markedly as to flg22^Rso^. The subsequent introduction of N significantly decreased flg22^Rso^-induced but not flg22^Pa^-induced ROS production. Further analysis revealed that the BAK1-interacting polymorphic residue H723R is primarily responsible for the suppressive effect of the N region, as introducing the R723H mutation into the chimera aaba increased ROS production in response to flg22^Rso^ (Extended Data Fig. 6a–c). However, the specific mechanisms by which P3 polymorphic residues influence flg22^Rso^ recognition remain unclear, as they are located in the C-terminal capping domain of the LRRs and the EJM domain (Fig. 3f)—regions that have not been previously studied. These findings underscore the significant role of ‘dark matter’ polymorphisms in modulating recognition specificity. The consistency between DNA shuffling predictions and subsequent experimental results highlights DNA shuffling as a powerful tool for exploring novel recognition specificities of PRRs.

By excluding the two identified suppressive regions and combining all ‘enhancer’ regions, we engineered a variant termed ‘superGmFLS2’, which exhibited a more than threefold increase in total ROS production in response to flg22^Rso^ compared to wild-type GmFLS2b (Fig. 4g and Extended Data Fig. 6d). There was also a slight enhancement in ROS production in response to flg22^Pa^ (Extended Data Fig. 6e), perhaps due to the P3 region discussed before (Extended Data Fig. 5k). The enhanced ROS production is not because of an increased FLS2 expression level (Extended Data Fig. 6g). However, we did not detect a significant increase in calcium influx (Extended Data Fig. 6f). Whether superGmFLS2 can confer stronger resistance to *R. solanacearum* than GmFLS2b remains to be tested in the future.

To test the hypothesis that aforementioned gain-of-function mutations enhance FLS2–BAK1 interaction to enable flg22^Rso^ recognition, we performed co-immunoprecipitation (co-IP) assays to assess NbBAK1 recruitment by GmFLS2a, GmFLS2a^Q600H, S603A^ and superGmFLS2 (Fig. 4h). Compared to GmFLS2a, both GmFLS2a^Q600H, S603A^ and superGmFLS2 exhibited enhanced NbBAK1 recruitment in response to both flg22^Rso^ and flg22^Pa^. Under mock treatment, a weak flg22-independent FLS2–BAK1 interaction was observed for superGmFLS2. These findings suggest that strengthening direct FLS2–BAK1 interaction could be a viable strategy for engineering recognition towards antagonistic flg22 variants.

### The broad applicability of FLS2 engineering principles

We then investigated whether the insights gained from reverse-engineering VrFLS2XL and GmFLS2b could be broadly applied to engineer FLS2s from other plants. Leveraging the modularity of our Golden Gate-based chimeric receptor construction strategy, we efficiently transplanted the LRR12-19 region from VrFLS2XL and the LRR20-EJM region from GmFLS2b into various FLS2 homologues irresponsive to flg22^Atum^ and flg22^Rso^ (Extended Data Figs. 1c–g and 8), replacing their native counterparts.

**Fig. 5:**
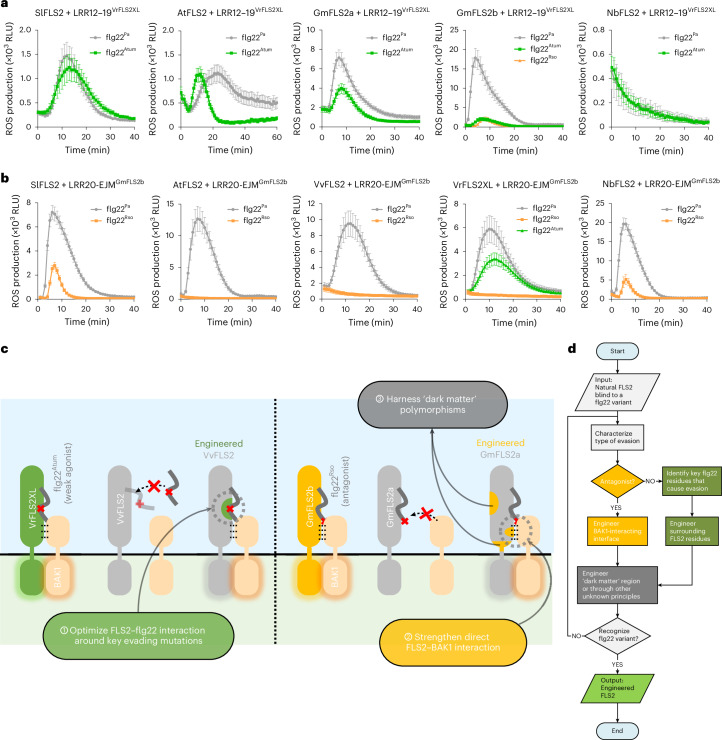
Summary of FLS2 engineering principles and their universal applicability. **a**, ROS responses of FLS2 homologues after transplantation of LRR12-19^VrFLS2XL^ (*n* = 24 leaf discs for SlFLS2 and GmFLS2a, and *n* = 12 leaf discs for AtFLS2, GmFLS2b and NbFLS2). **b**, ROS responses of FLS2 homologues after transplantation of LRR20-EJM^GmFLS2b^ (*n* = 36, 12, 24, 24, 12 leaf discs, from left to right). **c**, Summary of proposed design principles to engineer expanded recognition spectra towards evading flg22 variants: (1) Optimizing FLS2–flg22 interaction around key evasion sites to recognize weak agonistic flg22 variants with reduced binding affinity to FLS2. (2) Strengthening direct FLS2–BAK1 interaction to enable recognition of antagonistic flg22 variants that hinder BAK1 recruitment. (3) Harnessing dark matter polymorphisms that enhance performance through mechanisms that are not yet fully understood. **d**, Workflow for FLS2 engineering using these design principles. Based on the type of evasion—antagonism or loss of interaction with FLS2—appropriate design principles can be applied. Data are presented as mean ± s.e.m. All experiments were repeated at least three times with similar results. Source data

**Extended Data Fig. 7:**
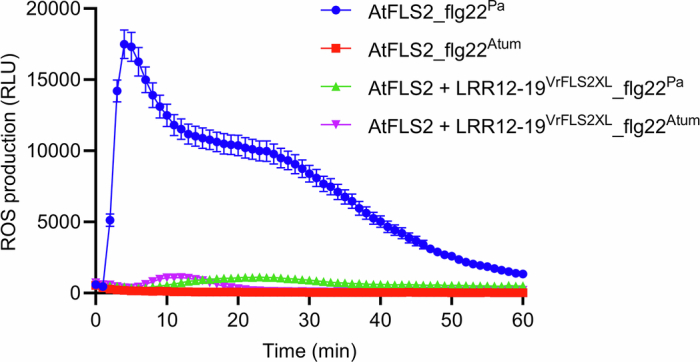
Flg22^Pa^- and flg22^Atum^-induced ROS responses of wild-type AtFLS2 and a chimeric AtFLS2 containing the LRR12-19 region from VrFLS2XL. Data are presented as mean ± SEM, with a sample size of *n* = 12 leaf discs. Similar results were obtained from three independent experiments. Source data

Transplanting LRR12-19^VrFLS2XL^ enabled AtFLS2, SlFLS2, GmFLS2a and GmFLS2b to gain recognition to flg22^Atum^. However, NbFLS2 lost its ability to detect canonical flg22^Pa^ following this transplantation (Fig. 5a). In addition, the ROS production kinetics induced by AtFLS2 with transplanted LRR12-19^VrFLS2XL^ exhibited unusual patterns: the flg22^Atum^-induced ROS burst rose and attenuated more rapidly than the flg22^Pa^-induced ROS burst, and both flg22^Pa^- and flg22^Atum^-induced responses were significantly weaker than those of wild-type AtFLS2 (Extended Data Fig. 7). This is possibly due to incompatibilities between residues from the two different origins at the junctions. It is worth noting that GmFLS2b with LRR12-19^VrFLS2XL^ maintained a reduced capability to recognize flg22^Rso^ while gaining recognition of flg22^Atum^. These findings suggest a generalizable design principle: engineering FLS2 recognition towards weak agonistic flg22 variants can be achieved by optimizing FLS2–flg22 interactions around key evasion sites (Fig. 5c, d).

**Extended Data Fig. 8:**
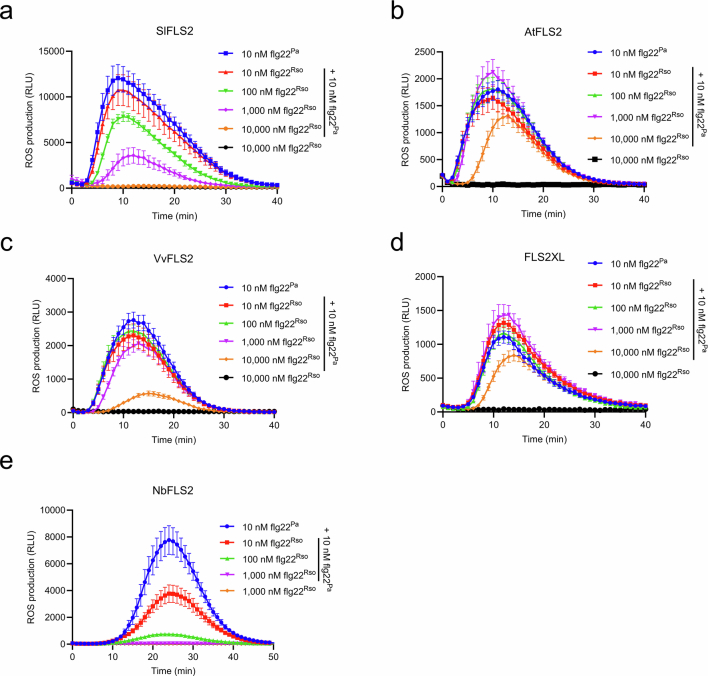
Evasion and antagonistic effect of flg22^Rso^ across FLS2 homologs. **a**, SlFLS2 (*n* = 8 leaf discs). **b**, AtFLS2 (*n* = 28 leaf discs). **c**, VvFLS2 (*n* = 24 leaf discs). **d**, VrFLS2XL (*n* = 24 leaf discs). **e**, NbFLS2 (*n* = 24 leaf discs). Data are presented as mean ± SEM. Similar results were obtained from two independent experiments. Source data

Transplantation of LRR20-EJM^GmFLS2b^ enabled SlFLS2 and NbFLS2 to recognize flg22^Rso^, but not AtFLS2, VvFLS2 and VrFLS2XL (Fig. 5b). To explain this, we assessed the evasion and antagonistic effects of flg22^Rso^ across different FLS2 orthologues (Extended Data Fig. 8). Flg22^Rso^ acted as a strong antagonist for SlFLS2 and NbFLS2 but showed minimal antagonistic effects for AtFLS2, VvFLS2 and VrFLS2XL. The correlation between the antagonism by flg22^Rso^ and the ability to gain flg22^Rso^ recognition after LRR20-EJM^GmFLS2b^ transplantation suggests that strengthening direct FLS2–BAK1 interaction is particularly effective for flg22 variants that function as strong antagonists. This finding also underscores the importance of first determining the evasion mechanism before initiating receptor engineering (Fig. 5c,d).

## Discussion

During plant–pathogen coevolution, plants have evolved PRRs with novel recognition specificities to detect PAMPs of pathogens, and pathogens have evolved polymorphic PAMPs to evade the recognition by PRRs. Editing native PRRs to gain recognition towards evading PAMPs offers a feasible strategy to generate durable broad-spectrum disease resistance without causing legal issues linked to transgene-based PRR transfer, but our knowledge on how to engineer such PRRs remains limited. In this study, we reverse-engineered two natural FLS2 variants and discovered fundamental design principles of broader recognition spectra against evading flg22 epitopes.

In our first reverse-engineering case using VrFLS2XL, we discovered that flg22 variants with reduced binding affinity to FLS2 can be addressed by optimizing the FLS2–flg22 interaction around key evasion sites of flg22. While this strategy seems straightforward and is supported by several studies^20,25^, it might not be easy to implement. Our results showed that approximately ten residue changes are required to make VvFLS2 strongly respond to flg22^Atum^ (Fig. 2h). Some of these residues are predicted to directly interact with key evasion sites of flg22^Atum^, some seem to interact with flg22^Atum^ residues adjacent to key evasion sites to assist with ligand binding, and others, including buried residues, appear to stabilize local conformation (Fig. 2b,c). It is worth noting that buried residues also play crucial roles, which are overlooked in Repeat Conservation Mapping analysis^49^ and previous attempts to engineer FLS2^20^. Moreover, the occasional simultaneous decreases in flg22^Pa^ and flg22^Atum^ responses observed in our mutation analysis (Extended Data Figs. 2b and 3a) suggest that the novel recognition specificity might come at the expense of existing recognition capabilities, although this could be rescued. Overall, the novel flg22^Atum^ recognition capability of VrFLS2XL appears to involve a complex and finely tuned interaction network. Therefore, further research is needed to elaborate this design principle to develop simpler solutions. In addition, while we derived this engineering strategy by studying the weakly agonistic flg22^Atum^, its applicability for engineering recognition of antagonistic flg22 variants remains to be investigated.

The reverse engineering of GmFLS2b offers a strategy to counteract the antagonistic flg22^Rso^ by strengthening the direct FLS2–BAK1 interaction without altering any flg22-interacting residues. It is worth noting that the gain of flg22^Rso^ recognition by GmFLS2a was achieved with the modification of only two residues (Fig. 4d,e and Extended Data Fig. 5), suggesting the effectiveness of this strategy. However, this strategy works only for FLS2 homologues to which flg22^Rso^ acts as an antagonist (Fig. 5b and Extended Data Fig. 8). Although this strategy was not reported in the initial GmFLS2b work^25^, the importance of FLS2–BAK1 direct interaction was suggested by several previous studies. For example, transplanting LRR19-24 of SlFLS2 to AtFLS2 enabled AtFLS2 to respond to flg22^Pa^ with 18AYA20 mutations^33^, and transplanting LRR18-24 of SlFLS2 to AtFLS2 led to AtBAK1-dependent autoactivation^50^. Future research should explore whether this principle applies to engineering recognition of antagonistic flg22 variants beyond flg22^Rso^. These findings also imply that the flg22-mediated and the direct FLS2–BAK1 interaction interfaces function coordinately rather than sequentially to recruit BAK1 as the reduced interaction at the first interface can be compensated by an increased interaction at the second interface. However, a potential risk of this approach is that enhancing direct FLS2–BAK1 interaction could also increase flg22-independent FLS2–BAK1 interaction (Fig. 4h), potentially triggering autoimmunity. This possibility should be further investigated using stable transgenic lines.

Using DNA shuffling, we discovered dark matter polymorphisms outside the flg22- or BAK1-interacting interfaces of GmFLS2a/b that significantly affect flg22^Rso^ recognition through unknown mechanisms, without altering FLS2 accumulation (Figs. 3c–e and 4c,f). The three asparagine-related polymorphisms within LRR12-19^GmFLS2b^ might lead to gains and losses in glycosylation. Although FLS2 has been shown to tolerate changes in glycosylation status^51–53^, it is possible that glycosylation subtly modulates FLS2’s recognition specificity through various mechanisms^54^. The six enhancer polymorphisms located in the C-terminal LRR capping domain and EJM domain point to the significance of this previously overlooked region, which aligns with earlier findings that mutations of cysteines within this region enhance FLS2-mediated ROS production^51^. Dark matter polymorphisms not only may influence FLS2 itself but could also affect its interaction with other positive or negative regulators, which could be further explored by immunoprecipitation–mass spectrometry assays in the future. Further investigation on dark matter polymorphisms will deepen our understanding of PRRs and may reveal general, ligand-independent design principles to engineer PRRs with enhanced functionality.

**Extended Data Fig. 9:**
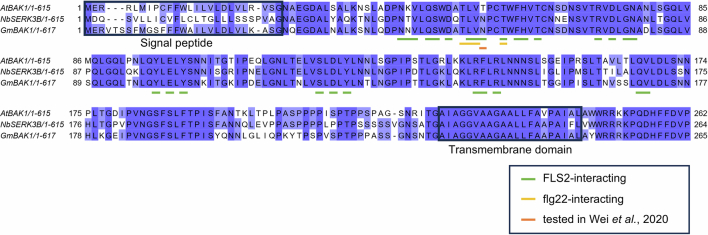
Alignment of BAK1 homologs. Annotations of FLS2- and flg22-interacting residues are based on ‘4MN8’ structure from the Protein Data Bank (PDB). At: *Arabidopsis thaliana*; Nb: *Nicotiana benthamiana*; Gm: *Glycine max*.

Although this study focuses on engineering FLS2, BAK1 presents an alternative target for expanding recognition spectra, particularly against those antagonistic flg22 variants. It is worth noting that co-expressing GmBAK1 with GmFLS2a has been reported to confer flg22^Rso^ recognition^25^, and our findings highlight the FLS2–BAK1 interface as a key engineering target. As shown in Extended Data Fig. 9, AtBAK1, NbBAK1 and GmBAK1 share high protein sequence identity (>80%). While residues directly interacting with FLS2 and flg22 are largely conserved, several polymorphic sites exist within and around the interface. The strategies used in this study to reverse-engineer FLS2 can be readily applied to GmBAK1 to identify and transfer key residues for flg22^Rso^ recognition. However, given BAK1’s role as a common co-receptor^55^, engineering BAK1 could have unintended consequences on plant immunity, growth and development.

From a methodological standpoint, our study establishes a pipeline for the identification of minimal sequence determinants responsible for novel recognition specificities in natural PRR variants. The three approaches used—domain swapping, structure-guided mutagenesis and DNA shuffling—each have their advantages and limitations, but they complement each other. Domain swapping is well suited for rough mapping of key residues. While finer mapping is possible by making more precise segmentations, this significantly increases the workload for molecular cloning. Computational design works best in cases where structures are resolved or predicted with high confidence. The more we understand about the PRR–ligand pair, the easier the mapping process becomes. However, this method may overlook crucial polymorphisms that are beyond current knowledge, as seen in the case of GmFLS2a/b. DNA shuffling, in other ways, enables rapid, unbiased fine mapping without much prior knowledge. However, it requires parental DNA fragments with high homology and polymorphic sites that are not tightly clustered^56^. For example, LRR20-EJM fragments of GmFLS2a and GmFLS2b are suitable candidates to shuffle, whereas LRR12-19 fragments of VvFLS2 and VrFLS2XL are not due to the large number of densely clustered polymorphic sites. Beyond these three approaches, other strategies can be used, such as leveraging coevolutionary insights^57^, ancestral sequence reconstruction^58^ and using Golden Gate shuffling that combines the advantages of domain swapping and DNA shuffling^59^. In addition, our study introduces an optimized method for constructing large, high-quality libraries of DNA variants directly in *A. tumefaciens*, using Golden Gate cloning and the *SacB* counterselection marker (Fig. 4a). However, the relatively low screening throughput remains a bottleneck, calling for technological innovation.

In addition to providing design principles for future FLS2 improvement, the engineering outcomes of this study already hold potential for practical applications. For example, to generate crown gall-resistant grapes, introducing 14 residue changes from the V2 list (Fig. 2a) into VvFLS2 requires 18 nucleotide substitutions, which is below the threshold of 20 nucleotides set by the current proposed European Union regulation on plants produced using new genomic techniques (COM/2023/411). Given that some V2 residues are dispensable for resistance (Fig. 2h and Extended Data Fig. 3c,d), the required modifications can be further minimized. These modifications could be introduced by prime editing and base editing, both of which are already available for grapevine^60,61^, or by homologous recombination^62^, which seems more straightforward but has not been reported for grapevine yet. Given that wild-type GmFLS2b has a relatively low recognition capacity for flg22^Rso^ compared to flg22^Pa^, yet still confers decent resistance against *R. solanacearum* when transiently expressed in roots under the control of the strong constitutive 35S promoter^25^, we assume that our superGmFLS2 engineered variant could confer an even stronger resistance when stably transformed into crops with an appropriate promoter. Alternatively, it could serve as a template for engineering native FLS2s in other crops to generate resistance to bacterial wilt disease.

Altogether, our study establishes fundamental principles for engineering expanded recognition spectra against two classic types of evading flg22 variant, provides a methodology for identifying sequence determinants of novel recognition specificity from natural PRR variants and offers ready-to-use genetic resources for developing resistance to crown gall disease and bacterial wilt. In the accompanying study, Li et al.^34^ used AlphaFold modelling and evolutionary analyses to highlight the importance of FLS2 residues interacting with polymorphic flg22 residues and the co-receptor in mediating expanded recognition specificity, supporting our findings. Combined with advancements in protein engineering techniques, genome editing technologies and evolving legal regulations as well as public acceptance towards genome-edited crops, our work paves the way for using PRR engineering to create gene-edited crops with durable broad-spectrum disease resistance.

## Methods

### Plant materials and synthetic peptides

Five-week-old *N. benthamiana fls2* mutant^26^ was used for ROS burst assays and infection assays with *A. tumefaciens*. Transgenic *N. benthamiana* expressing aequorin^63^ was used for cytoplasmic calcium measurement. *N. benthamiana* plants were grown in the greenhouse at 60% relative humidity and 25 °C/22 °C in a 16 h/8 h day/night cycle. Synthetic peptides of flg22^Pa^, flg22^Atum^ and flg22^Rso^ (sequences shown in main figures) were produced by Scilight Biotechnology at >80% purity. Peptide powder was dissolved in sterile ddH_2_O to a concentration of 10 mM as a stock solution and stored at −20 °C. Working solution with a certain concentration was freshly diluted from the stock solution before use. Unless specified, 100 nM of elicitor peptide was used to trigger immune responses.

### Transient expression in *N. benthamiana*

*A. tumefaciens* GV3101 transformed with certain binary plasmid was grown overnight in liquid Luria–Bertani (LB) medium supplemented with gentamycin, rifampicin and antibiotics corresponding to the binary vector (spectinomycin for FLS2 plasmids, kanamycin for P19 and GUS plasmids). Cells were collected by centrifugation (3,000 × *g*, 5 min), washed once and resuspended in the infiltration buffer (10 mM MES–KOH, pH 5.8, 10 mM MgCl_2_, 150 μM acetosyringone). Cell density (optical density at 600 nm (OD_600_)) was measured using a Bio-Rad SmartSpec Plus spectrophotometer. For infiltration, *A. tumefaciens* carrying the FLS2 plasmid and a second strain carrying the P19 plasmid were mixed at a 4:1 ratio to reach a final OD_600_ of 0.5 (0.4 FLS2 + 0.1 P19). For quantitative comparison of two FLS2 variants, two freshly transformed *A. tumefaciens* strains carrying two constructs were symmetrically infiltrated into two sides of the midrib. For Extended Data Fig. 4e–g, chimera abbb was co-infiltrated at OD_600_ values of 0/0.1/0.2/0.4/0.8 along with P19 (OD_600_ = 0.1) and untransformed GV3101 at OD_600_ values of 0.8/0.7/0.6/0.4/0, respectively, to maintain a constant final OD_600_ of 0.9 across all conditions.

### Molecular cloning

To generate FLS2 chimeras and mutants in a modular manner, a unified multi-kingdom Golden Gate cloning platform was used^38,64^. Template plasmids of NbFLS2, AtFLS2 and VvFLS2 were from the lab stock. VrFLS2XL and SlFLS2 were gifts from G. Felix (University of Tübingen). GmFLS2a and GmFLS2b were gifts from A. Macho (Chinese Academy of Sciences). N-LRR11, LRR12-19, LRR20-EJM and TMD + ICD segments of aforementioned FLS2s were amplified from template plasmids and subcloned to p641-Esp3I/BpiI level 1 entry vectors. To assemble FLS2 segments, a level 2 receiver plasmid was constructed by assembling a 35S promoter (Addgene 54406), a *LacZ* expression cassette flanked by Esp3I sites (Addgene 54458), a C-terminal mEGFP tag (Addgene 54410 with A206K mutation), a NOS terminator (Addgene 54407) and dummies (Addgene 54342, 54345) into a level 2 binary vector (Addgene 54346). LRR12-19^VvFLS2^ fragments carrying computationally predicted polymorphic residue sets (V1, V2, V3), the LRR20-EJM^GmFLS2a^ fragment carrying YHR and YHAIR (F579Y, Q600H, S603A, M626I, H723R) mutations and the codon-optimized LRR20-EJM^GmFLS2b^ fragment for DNA shuffling were synthesized by Twist Bioscience. To construct the receiver plasmid for shuffled DNA fragments, the *SacB* expression cassette was first cloned into the p641-BpiI level 1 vector using pT18mobsacB (Addgene 72648) as PCR template, meanwhile introducing flanking Esp3I sites and the same overhangs as the LRR20-EJM fragment. Then, the level 2 receiver plasmid was constructed by assembling level 1 plasmids of 35S promoter, NOS terminator, C-terminal mEGFP tag, N-LRR11^GmFLS2a^, LRR12-19^GmFLS2a^, TMD+ICD^GmFLS2a^, *SacB* expression cassette and dummies. Site-directed mutagenesis was conducted in level 1 plasmid using a Golden Gate-based strategy^65^. The level 2 NbBAK1 expression plasmid for co-IP was constructed by assembling a 35S promoter, a NbBAK1 coding sequence, a C-terminal 4×HA tag, a NOS terminator and dummies. The template plasmid used to amplify NbBAK1 and the level 1 entry plasmid containing C-terminal 4×HA tag were from lab stock. DNA sequences are listed in Supplementary Table 1. Primer sequences are listed in Supplementary Table 2.

### Measurement of ROS production

At least eight leaf discs per treatment were collected from each leaf area transiently expressing a certain FLS2 variant; these were collected with a Ø 4 mm biopsy punch (KAI, BP-40F) and floated on 100 μl distilled water overnight in white 96-well plates (Greiner Bio-One, 655075) in darkness. Immediately after replacing water with ROS assay solution (100 μM luminol (Sigma, A8511), 20 μg ml^−1^ horseradish peroxidase (Sigma, 77332)) with or without elicitors, luminescence was recorded as relative light units (RLU) using a microplate reader (Tecan Spark, Berthold Tristar 3 or Tecan Infinite Lumi) with 1 min intervals and 250 ms integration time. At least three different leaves from three plants were sampled for each FLS2 variant. Unless specified, total ROS production over 40 min was calculated. In the antagonism assay, flg22^Pa^ and flg22^Rso^ were co-administered at the same time.

### Cytoplasmic calcium measurement

At least eight leaf discs per treatment were collected from each leaf area transiently expressing a certain FLS2 variant using a Ø 4 mm biopsy punch (KAI, BP-40F) and incubated in 100 μl of 10 μM coelenterazine water solution in darkness overnight in white 96-well plates (Greiner Bio-One, 655075). After replacing the coelenterazine solution with 75 μl of distilled water, luminescence was measured using a Tecan Spark multimode microplate reader. As a baseline, luminescence was first recorded in 30 s intervals for 5 min. Then, 25 μl of water (as mock) or a four-time concentrated elicitor water solution was added to each well, and luminescence was recorded in 30 s intervals for 30 min. The remaining aequorin signal was discharged by adding 100 μl of discharge solution (2 M CaCl_2_, 20% EtOH) and measured for 90 s. A normalized signal ‘*L*/*L*_max_’ was calculated for each time point, where ‘*L*’ is the recorded absolute luminescence at each time point and ‘*L*_max_’ is the total luminescence during 90 s discharging.

### Protein extraction and western blot

For total protein extraction to check protein expression levels, *N. benthamiana* leaf tissues were sampled into 2 ml tubes with Ø 4 mm glass beads, snap-frozen in liquid nitrogen, grounded in SPEX SamplePrep 2010 Geno/Grinder and homogenized in protein extraction buffer (50 mM Tris–HCl pH 7.5, 100 mM NaCl, 10% glycerol, 2 mM EDTA, 5 mM DTT, 1% IGEPAL CA630, 2 mM sodium molybdate, 1 mM sodium fluoride, 1 mM sodium orthovanadate, 4 mM sodium tartrate, protease inhibitor cocktail). Samples were incubated at 4 °C for 10–20 min and then centrifuged at 16,000 × *g* for 20 min at 4 °C to remove debris. Supernatants were mixed with SDS loading buffer and boiled at 95 °C for 5 min before loading. Proteins were separated using 10% SDS–PAGE gels and transferred to a PVDF membrane. The membrane was blocked using 5% skim milk in TBST and probed using the corresponding antibodies diluted in TBST–5% skim milk. Blotted membranes were stained with Coomassie brilliant blue as loading control. To detect the expression levels of FLS2 variants, GFP Antibody (B-2) (Santa Cruz, sc-9996, 1:5,000) was used as the primary antibody, and Anti-Mouse IgG (Fc specific)–Peroxidase (Sigma-Aldrich, A0168, 1:15,000) was used as the secondary antibody.

### Detection of MAPK phosphorylation

After transiently expressing FLS2 variants for 3 days, intact *N. benthamiana* leaves were syringe-infiltrated with 100 μM flg22^Rso^ or ddH_2_O only as a mock control. After flg22^Rso^ treatment for 15 min, eight leaf discs were collected into 2 ml tubes containing five Ø 4 mm glass beads using a Ø 8 mm biopsy punch (KAI, BP-80F). Protein extraction, SDS–PAGE and western blot were performed as aforementioned, but 5% bovine serum albumin in TBST was used for blocking and diluting antibodies. Phospho-p44/42 MAPK (Erk1/2) (Thr202/Tyr204) antibody (Cell Signaling Technology, 9101, 1:1,000) and Anti-Rabbit IgG (whole molecule)–Peroxidase (Sigma-Aldrich, A0545, 1:10,000) were used to detect phosphorylated MAPKs.

### Co-IP

Each FLS2 mutant was co-agroinfiltrated with BAK1 and P19 into 12 half-leaves using an OD_600_ of 0.5 for FLS2, 0.5 for BAK1 and 0.1 for P19. Three days after infiltration, leaves were collected and midribs removed. For each treatment, four half-leaves per FLS2 mutant were used. Elicitor treatments were performed by vacuum-infiltrating the leaves with 100 nM elicitor solution for 3 min, followed by pressure release. Distilled water served as the mock treatment. Vacuum infiltration was repeated until the leaves were fully infiltrated. Fifteen minutes after the start of the first vacuum infiltration, leaves were blotted dry with paper towels and snap-frozen in liquid nitrogen. Frozen leaf tissue was ground to a fine powder using a liquid nitrogen-cooled mortar and pestle. Extraction buffer was added to the ground tissue at a volume ratio of 1.2:1, and proteins were solubilized by rotating the mixture at 4 °C for 30 min. Extracts were filtered through two layers of Miracloth and centrifuged at 25,000 × *g* for 30 min at 4 °C to collect the supernatant. Protein concentrations were estimated using the Bradford assay, and extracts were normalized before incubation with 20 μl of ChromoTek GFP-Trap Agarose for 2 h at 4 °C on a rotator. Samples were then centrifuged at 1,000 × *g* for 2 min at 4 °C using a tabletop centrifuge, and the supernatant was removed. Beads were washed four times with 1 ml of extraction buffer. For western blot analysis, agarose beads were resuspended in 50 μl of 2× SDS–PAGE loading buffer and heated at 90 °C for 10 min. SDS–PAGE and western blot were performed as aforementioned. NbBAK1-HA was detected using anti-HA-HRP antibody (Roche, 12013819001, 1:2,000).

### Measurement of GUS activity by histochemical and fluorometric assays

As shown in Extended Data Fig. 1g, after transiently expressing FLS2 variants for 3 days, whole *N. benthamiana* leaves were infiltrated with *A. tumefaciens* GV3101 carrying pBIN19g:GUS (intronic) plasmid at OD_600_ = 0.5 (suspended in infiltration buffer without acetosyringone). For the histochemical GUS assay, 4 days after infiltration, the whole leaves were collected and vacuum-infiltrated thoroughly in GUS staining solution (0.1 M sodium phosphate buffer pH 7.0, 10 mM EDTA, 0.1% Triton X-100, 1 mM K_3_[Fe(CN)_6_], 2 mM X-Gluc). After overnight incubation in GUS staining solution at 37 °C, samples were destained in 70% ethanol at 60 °C before taking photos. For the fluorometric GUS assay, 4 days after infiltration, six leaf discs for each FLS2 variant were sampled in 2 ml tubes containing three Ø 4 mm glass beads using a Ø 4 mm biopsy punch (KAI, BP-40F) and were snap-frozen in liquid nitrogen. Subsequent steps were performed as previously described^66^.

### Quantification of *A. tumefaciens* population size

As shown in Extended Data Fig. 1g, after transiently expressing FLS2 variants for 3 days, whole *N. benthamiana* leaves were infiltrated with either *A. tumefaciens* GV3101 carrying pBIN19g:GUS (intronic) plasmid (for colony counting) or AgroLux strain^41^ (for bioluminescence measurement) at OD_600_ = 0.5 (suspended in infiltration buffer without acetosyringone). Four days after infiltration, bacterial population sizes were quantified. For colony counting, three 8-mm-diameter leaf discs were collected per sample and ground in 200 µl of 10 mM MgCl_2_ solution (adaptor and solution were pre-cooled at 4 °C to prevent the heat stress generated during homogenization). The volume was then adjusted to 1 ml by adding 800 µl of 10 mM MgCl_2_. The resulting extracts were serially diluted and plated onto LB agar plates containing kanamycin, gentamicin and rifampicin. After incubation for 48 h at 28 °C, colony numbers were counted. Data were obtained from six independent biological replicates for statistical analysis. AgroLux luminescence was measured using a Tecan Spark microplate reader for 15 min with 1 min intervals and 500 ms integration time. Data can be recorded when the curves become stable. At least eight leaf discs from each leaf and three different leaves from three plants were sampled for each FLS2 variant.

### Prediction, visualization and analysis of protein complex structure

The structure of the FLS2–flg22–BAK1 complex was predicted using AlphaFold3 server^37^. Signal peptides were removed from FLS2 and BAK1 before uploading sequences for prediction. The top-ranked (by the interface predicted template modelling (ipTM) scores) models were further analysed using Mapiya^67^ to determine all possible interactions, for example, hydrogen bonds, salt bridges, hydrophobic contacts, electrostatic interactions (dipole–dipole, dipole–π stacking), within 4 and 8 Å between FLS2 and flg22 or FLS2 and BAK1. The predicted models and interactive residues were visualized by PyMol version 1.8 (ref. ^68,69^).

### DNA shuffling and direct variant library construction in *A. tumefaciens*

For details and tips to plan a DNA shuffling experiment, please refer to Meyer et al.^56^. Parental DNA fragments for DNA shuffling were prepared by amplifying level 1 entry plasmids carrying LRR20-EJM^GmFLS2a^ and LRR20-EJM^GmFLS2b^ using the outer primer pair SZ172 and SZ173, and purifying PCR product from the 1% agarose gels. For an optimal DNA shuffling outcome, LRR20-EJM^GmFLS2b^ was codon-optimized to achieve maximum homology with LRR20-EJM^GmFLS2a^. DNA Shuffling was performed using the JBS DNA-Shuffling Kit (Jena Bioscience, PP-103). Shuffled DNA fragments were amplified using the inner primer pair SZ621 and SZ622. The PCR product was purified and ligated into the *SacB*-containing vector by Golden Gate cloning. Golden Gate product was purified using the DNA Clean & Concentrator-5 kit (Zymo Research, D4003) and transformed to *A. tumefaciens* GV3101 by electroporation. Transformed cells were plated on LB plates supplemented with gentamycin, rifampicin, spectinomycin and 5% sucrose. Single colonies were cultured in 96-well plates and infiltrated to *N. benthamiana fls2* mutant. Meanwhile, colonies were sequenced. Primer sequences are listed in Supplementary Table 2.

### Analysis of DNA shuffling results

The performance of a shuffled variant is defined by the ratio of the peak values (RLU_max_) of flg22^Rso^- and flg22^Pa^-induced ROS curves. Variants showing no visible flg22^Rso^-induced ROS burst were assigned a performance of zero. The performance of a residue at a polymorphic site is calculated as the average performance of all variants carrying this residue. For example, the performance of alanine from GmFLS2a at position 536 is calculated by averaging the performances of all shuffled variants with an alanine at position 536. A Welch’s *t*-test (unequal variances *t*-test) is conducted for each polymorphic site to compare the mean performance of residue from GmFLS2b against that from GmFLS2a. A high *T*-statistic absolute value suggests a significant difference between the performances of residues sourced from GmFLS2a and GmFLS2b at that position, implying that this site might be important for flg22^Rso^ recognition. Instead, a low *T*-statistic absolute value suggests that this site might not be important for flg22^Rso^ recognition. A positive *T*-statistic value means that the residue of GmFLS2b origin at this position positively contributes to flg22^Rso^ recognition. A negative *T*-statistic value means that the residue of GmFLS2a origin at this position negatively contributes to flg22^Rso^ recognition.

### Statistical analysis

Statistical analyses were performed using GraphPad Prism 10.1.2 (324). Data normality was assessed with the Anderson–Darling test or Kolmogorov–Smirnov test (if sample size is too small to use Anderson–Darling test). For data following a normal distribution, two-group comparisons were conducted using a two-tailed Student’s *t*-test, while multiple comparisons were analysed using one-way analysis of variance (ANOVA) followed by Tukey’s test. For data not following a normal distribution, two-group comparisons were performed using a two-tailed Mann–Whitney test, and multiple comparisons were assessed with the Kruskal–Wallis test followed by Dunn’s post hoc test. Reported *P* values reflect analyses conducted using the two-tailed Student’s *t*-test or the two-tailed Mann–Whitney test. Different letters indicate statistically significant differences as determined by one-way ANOVA with Tukey’s test or Kruskal–Wallis with Dunn’s test (*P* < 0.05). Exact *P* values are provided in the Source data. The number of replicates is provided in the figure legends. For box plots: centre line, median; box limits, upper and lower quartiles; whiskers, 1.5 times interquartile range; points, outliers.

### Reporting summary

Further information on research design is available in the Nature Portfolio Reporting Summary linked to this article.

## Supplementary information

Reporting Summary

Supplementary Table 1 DNA sequences used in this study.

Supplementary Table 2 Primers used in this study.

Supplementary Table 3 Shuffled GmFLS2a/b LRR20-EJM variants used for analysis.

Supplementary Data 1 AlphaFold3-predicted VrFLS2XL-flg22^Atum^-NbBAK1 complex structure.

Supplementary Data 2 AlphaFold3-predicted GmFLS2b-flg22^Rso^-NbBAK1 complex structure.

## Source data

Source Data Figures and Extended Data Figures Statistical source data.

Source Data Figures and Extended Data Figures Unprocessed images of western blots and histochemical GUS assay.

## Data Availability

All data supporting the findings of this study are available within the paper and its Supplementary Information. The crystal structure of *A. thaliana* FLS2–flg22–BAK1 complex analysed during the current study is available in the Protein Data Bank with accession code 4MN8 (https://www.rcsb.org/structure/4MN8). Source data are provided with this paper.
