# Author Correction: Reverse engineering of the pattern recognition receptor FLS2 reveals key design principles of broader recognition spectra against evading flg22 epitopes

**DOI:** 10.1038/s41477-025-02166-8

**Published:** 2025-11-06

**Authors:** Songyuan Zhang, Songyuan Liu, Hung-Fei Lai, Kyle W. Bender, Gijeong Kim, Amedeo Caflisch, Cyril Zipfel

**Affiliations:** 1 Institute of Plant and Microbial Biology, Zurich-Basel Plant Science Center, University of Zurich, Zurich, Switzerland; 2 Department of Biochemistry, University of Zurich, Zurich, Switzerland; 3 The Sainsbury Laboratory, University of East Anglia, Norwich Research Park, Norwich, UK

**Keywords:** Pattern recognition receptors in plants, Plant signalling, Molecular engineering in plants

Correction to: *Nature Plants* 10.1038/s41477-025-02050-5, published online 28 July 2025.

In the version of the article initially published, in Fig. 4h, the “α-HA” image was a duplicate of the “α-GFP” image. The correct “α-HA” image has now been added to Fig. 4h, and the corresponding source data, in the HTML and PDF versions of the article.

