# Supplementary material for: Reverse engineering of the pattern recognition receptor FLS2 reveals key design principles of broader recognition spectra against evading flg22 epitopes
Source: Nat Plants. 2025 Jul 28;11(8):1642–57. doi: 10.1038/s41477-025-02050-5 (PMC12364711; doi:10.1038/s41477-025-02050-5)
Supplement: Supplementary file 1 — Reporting Summary [file 41477_2025_2050_MOESM1_ESM.pdf]

# nature portfolio

Corresponding author(s): Cyril Zipfel

Last updated by author(s): Jun 3, 2025

# Reporting Summary

Nature Portfolio wishes to improve the reproducibility of the work that we publish. This form provides structure for consistency and transparency in reporting. For further information on Nature Portfolio policies, see our Editorial Policies and the Editorial Policy Checklist.

## Statistics

For all statistical analyses, confirm that the following items are present in the figure legend, table legend, main text, or Methods section.

| n/a | Confirmed | |
|---|---|---|
| ☐ | ☒ | The exact sample size (*n*) for each experimental group/condition, given as a discrete number and unit of measurement |
| ☐ | ☒ | A statement on whether measurements were taken from distinct samples or whether the same sample was measured repeatedly |
| ☐ | ☒ | The statistical test(s) used AND whether they are one- or two-sided<br>*Only common tests should be described solely by name; describe more complex techniques in the Methods section.* |
| ☒ | ☐ | A description of all covariates tested |
| ☐ | ☒ | A description of any assumptions or corrections, such as tests of normality and adjustment for multiple comparisons |
| ☐ | ☒ | A full description of the statistical parameters including central tendency (e.g. means) or other basic estimates (e.g. regression coefficient) AND variation (e.g. standard deviation) or associated estimates of uncertainty (e.g. confidence intervals) |
| ☐ | ☒ | For null hypothesis testing, the test statistic (e.g. *F*, *t*, *r*) with confidence intervals, effect sizes, degrees of freedom and *P* value noted<br>*Give P values as exact values whenever suitable.* |
| ☒ | ☐ | For Bayesian analysis, information on the choice of priors and Markov chain Monte Carlo settings |
| ☒ | ☐ | For hierarchical and complex designs, identification of the appropriate level for tests and full reporting of outcomes |
| ☒ | ☐ | Estimates of effect sizes (e.g. Cohen's *d*, Pearson's *r*), indicating how they were calculated |

*Our web collection on statistics for biologists contains articles on many of the points above.*

## Software and code

Policy information about availability of computer code

| | |
|---|---|
| Data collection | SparkControl and i-Control (Tecan); LightCompass 6.16 (Berthold); ChemiDoc (Bio-Rad); AlphaFold Server |
| Data analysis | GraphPad Prism 10.1.2 (324); Jalview 2.11.4.1; PyMol 1.8; MAPIYA contact map server |

For manuscripts utilizing custom algorithms or software that are central to the research but not yet described in published literature, software must be made available to editors and reviewers. We strongly encourage code deposition in a community repository (e.g. GitHub). See the Nature Portfolio guidelines for submitting code & software for further information.

## Data

Policy information about availability of data

All manuscripts must include a data availability statement. This statement should provide the following information, where applicable:

- Accession codes, unique identifiers, or web links for publicly available datasets
- A description of any restrictions on data availability
- For clinical datasets or third party data, please ensure that the statement adheres to our policy

All data supporting the findings of this study are available within the paper and its Supplementary Information. The crystal structure of Arabidopsis thaliana FLS2-flg22-BAK1 complex analyzed during the current study is available in the Protein Data Bank with accession code 4MN8 (https://www.rcsb.org/structure/4MN8). Original western blot images, data points in graphs, and statistical analysis results are provided in the source data.

# Research involving human participants, their data, or biological material

Policy information about studies with human participants or human data. See also policy information about sex, gender (identity/presentation), and sexual orientation and race, ethnicity and racism.

| | |
|---|---|
| Reporting on sex and gender | No human data is involved in this research. |
| Reporting on race, ethnicity, or other socially relevant groupings | No human data is involved in this research. |
| Population characteristics | No human data is involved in this research. |
| Recruitment | No human data is involved in this research. |
| Ethics oversight | No human data is involved in this research. |

Note that full information on the approval of the study protocol must also be provided in the manuscript.

# Field-specific reporting

Please select the one below that is the best fit for your research. If you are not sure, read the appropriate sections before making your selection.

☒ Life sciences ☐ Behavioural & social sciences ☐ Ecological, evolutionary & environmental sciences

For a reference copy of the document with all sections, see nature.com/documents/nr-reporting-summary-flat.pdf

# Life sciences study design

All studies must disclose on these points even when the disclosure is negative.

| | |
|---|---|
| Sample size | Sample sizes are detailed in the figure captions. They were selected to be as large as practically possible and were guided by established protocols from previous studies: ROS assay (DOI: 10.1007/978-1-4939-7063-6_7); calcium influx measurement (DOI: 10.1104/pp.110.171249); GUS assay (DOI: 10.7554/eLife.92110); and AgroLux imaging and CFU counting (DOI: 10.1111/tpj.15454). |
| Data exclusions | Data were excluded if leaf disc samples were accidentally damaged during the assays. As the sample sizes were sufficiently large and the experiments were repeated multiple times, these exclusions are not expected to impact the overall conclusions. Exclusion criteria were pre-established. |
| Replication | All experiments were repeated independently for at least three times with similar results, except for Fig. 1h, Fig, 2g, Fig. 4h, Extended Data Figs. 1k, 2c, 3b-d, 4d-g, 5i, which were repeated twice, as described in figure captions. |
| Randomization | All infiltrated plants were randomly allocated to minimize environmental variation. Leaf discs were randomly collected from the infiltrated areas, avoiding veins and edges of infiltrated areas. |
| Blinding | Blinding was not applicable to this study. All the experiments were carried out without prior knowledge of the the final outcomes. |

# Reporting for specific materials, systems and methods

We require information from authors about some types of materials, experimental systems and methods used in many studies. Here, indicate whether each material, system or method listed is relevant to your study. If you are not sure if a list item applies to your research, read the appropriate section before selecting a response.

## Materials & experimental systems

| n/a | Involved in the study | |
|---|---|---|
| ☐ | ☒ | Antibodies |
| ☒ | ☐ | Eukaryotic cell lines |
| ☒ | ☐ | Palaeontology and archaeology |
| ☒ | ☐ | Animals and other organisms |
| ☒ | ☐ | Clinical data |
| ☒ | ☐ | Dual use research of concern |
| ☐ | ☒ | Plants |

## Methods

| n/a | Involved in the study | |
|---|---|---|
| ☒ | ☐ | ChIP-seq |
| ☒ | ☐ | Flow cytometry |
| ☒ | ☐ | MRI-based neuroimaging |

## Antibodies

| | |
|---|---|
| Antibodies used | GFP Antibody (B-2) (Santa Cruz, #sc-9996, 1:5,000); Anti-Mouse IgG (Fc specific)-Peroxidase (Sigma-Aldrich, #A0168, 1:15,000); Phospho-p44/42 MAPK (Erk1/2) (Thr202/Tyr204) antibody (Cell Signaling Technology, #9101, 1:1,000); Anti-Rabbit IgG (whole molecule)–Peroxidase (Sigma-Aldrich, #A0545, 1:10,000); Anti-HA-HRP antibody (Roche, #12013819001, 1:2,000) |
| Validation | Validation statements are available from the corresponding manufactures' websites.<br>GFP Antibody (B-2): https://www.scbt.com/p/gfp-antibody-b-2<br>Anti-Mouse IgG (Fc specific)-Peroxidase: https://www.sigmaaldrich.com/CH/en/product/sigma/a0168<br>Phospho-p44/42 MAPK (Erk1/2) (Thr202/Tyr204) antibody: https://www.cellsignal.com/products/primary-antibodies/p44-42-mapk-erkl-2-antibody/9102<br>Anti-Rabbit IgG (whole molecule)–Peroxidase: https://www.sigmaaldrich.com/CH/en/product/sigma/a0545<br>Anti-HA-HRP antibody: https://www.sigmaaldrich.com/CH/en/product/roche/12013819001 |

## Plants

| | |
|---|---|
| Seed stocks | The Nicotiana benthamiana fls2 mutant line was obtained from Qiang Cheng and was previously reported (DOI: 10.1111/pbi.13898).<br>The N. benthamiana aequorin line was from the lab stock and was previously reported (DOI: 10.1104/pp.110.171249). |
| Novel plant genotypes | No novel plant genotype was created in this study. |
| Authentication | The flg22-induced ROS burst and cytoplasmic calcium influx were measured as described in the Methods section to confirm the identities of the Nicotiana benthamiana fls2 mutant line and the aequorin line, respectively. |
