# Supplementary material for: Reverse engineering of the pattern recognition receptor FLS2 reveals key design principles of broader recognition spectra against evading flg22 epitopes
Source: Nat Plants. 2025 Jul 28;11(8):1642–57. doi: 10.1038/s41477-025-02050-5 (PMC12364711; doi:10.1038/s41477-025-02050-5)

Fig. 1f

the same blot merged with molecular weight marker
but with different exposure time

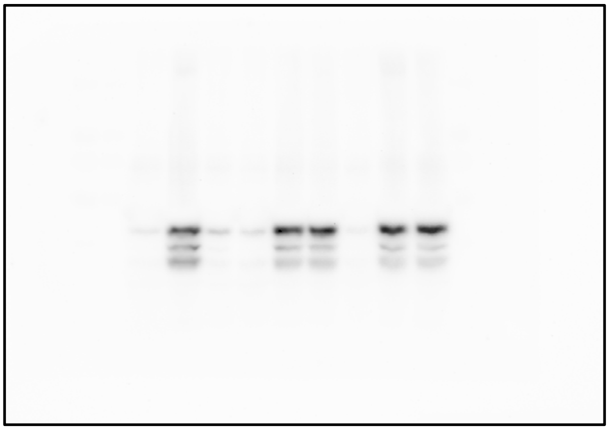

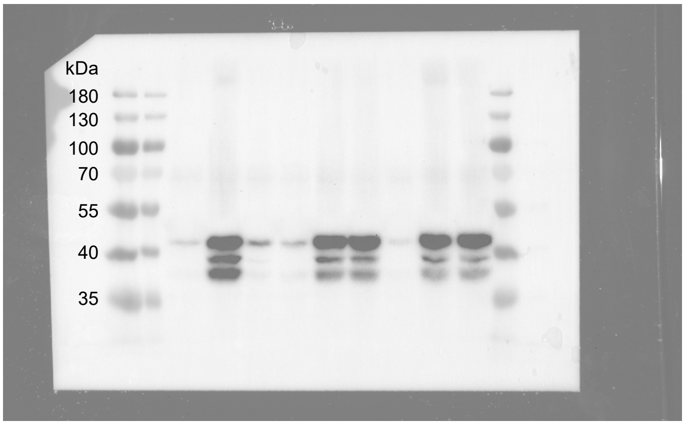

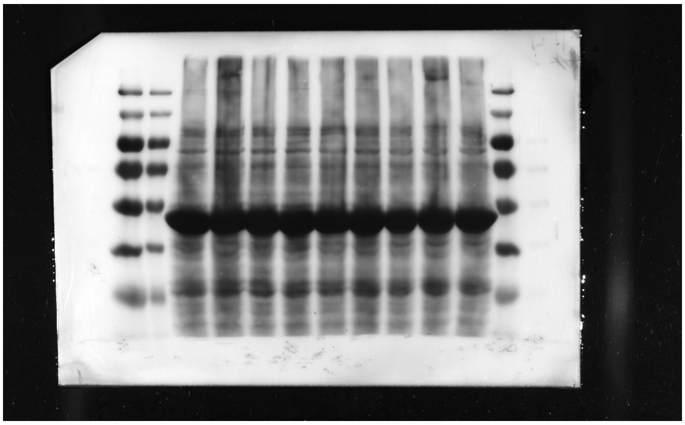

Fig. 1g

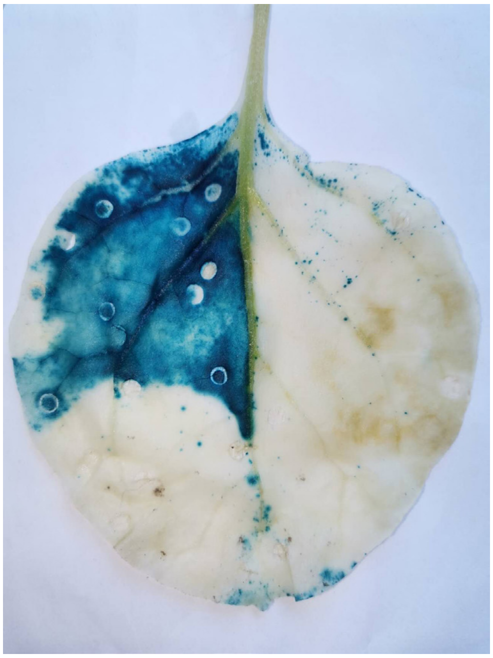

Fig. 2e

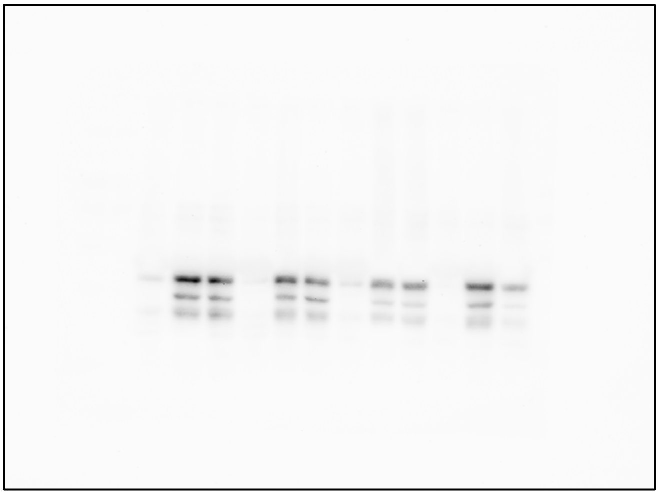

the same blot merged with molecular weight marker but with different exposure time

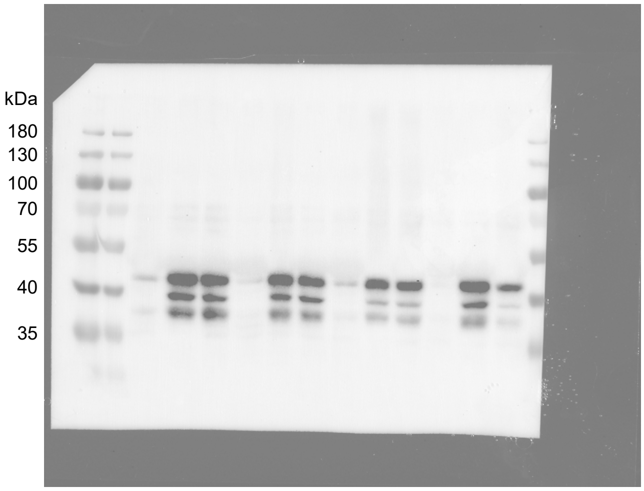

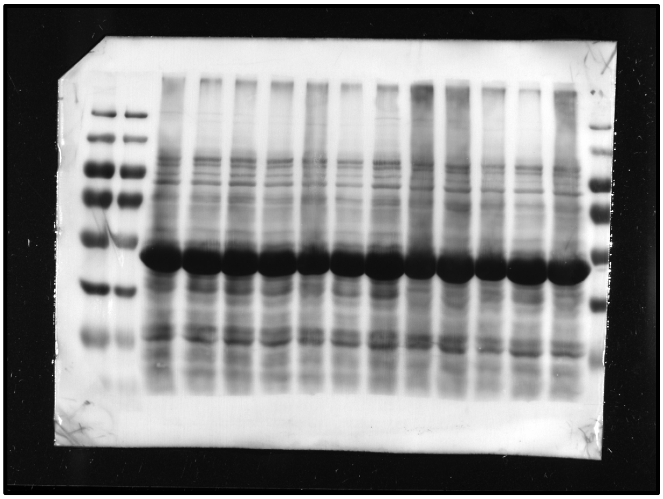

Fig. 4h

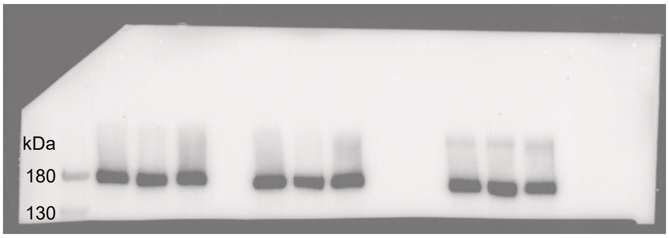

the same blot merged with molecular weight marker but with different exposure time

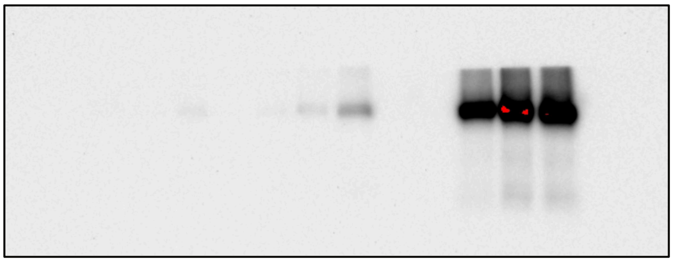

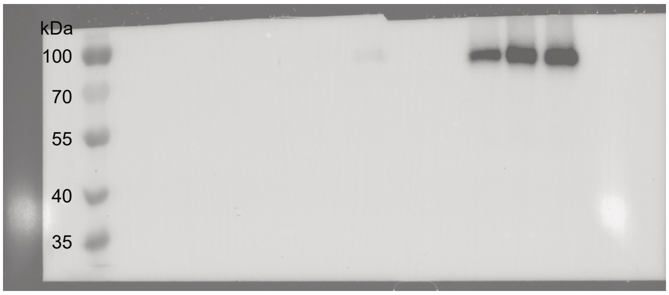

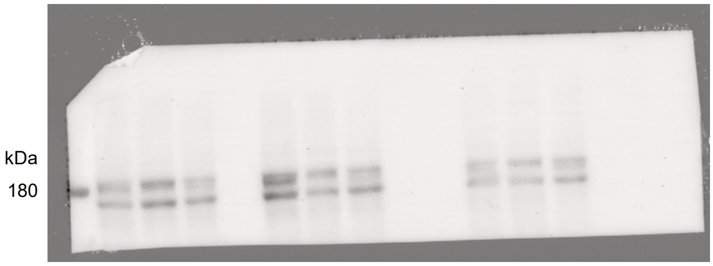

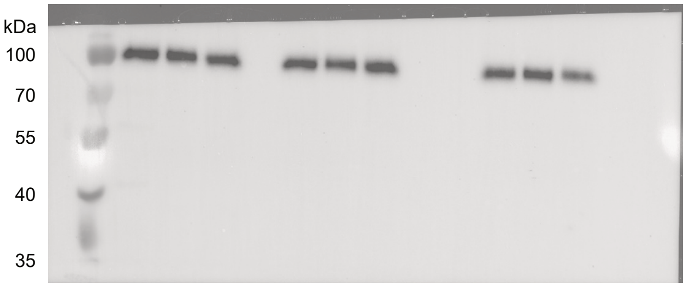

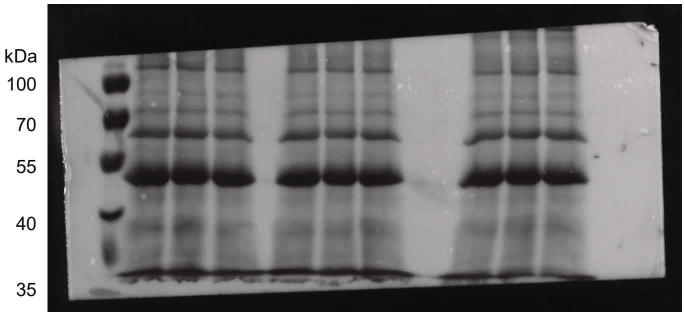

Extended Data Fig. 2d

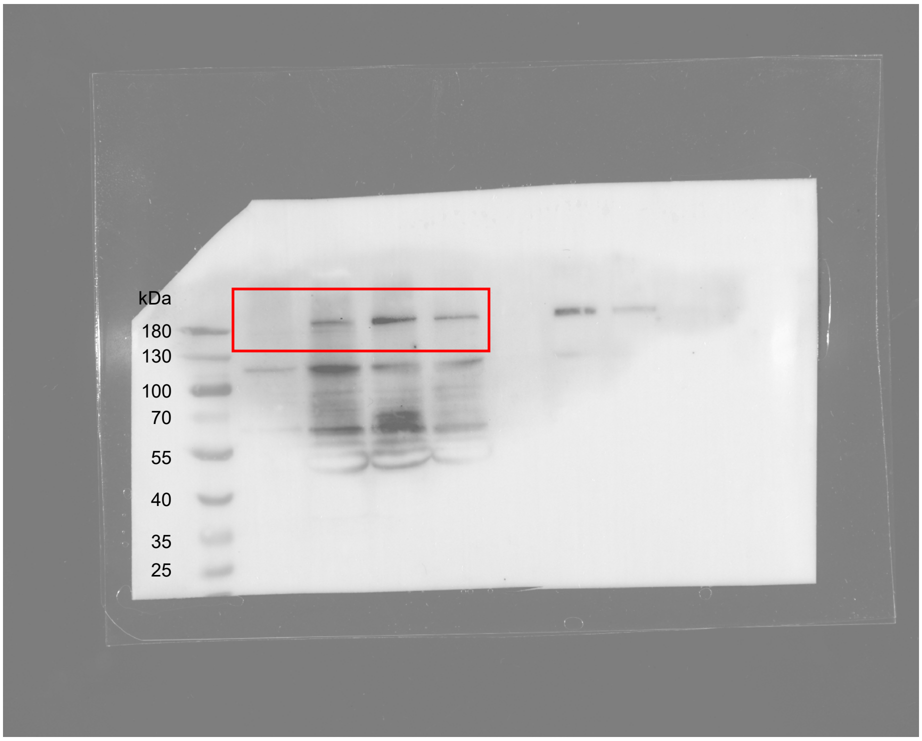

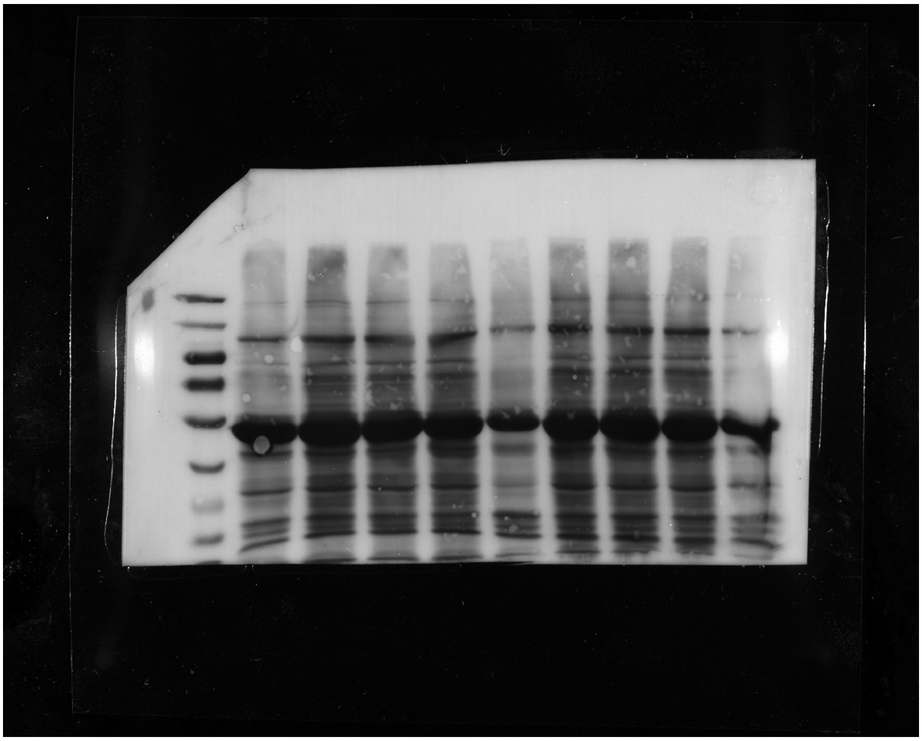

Extended Data Fig. 3b

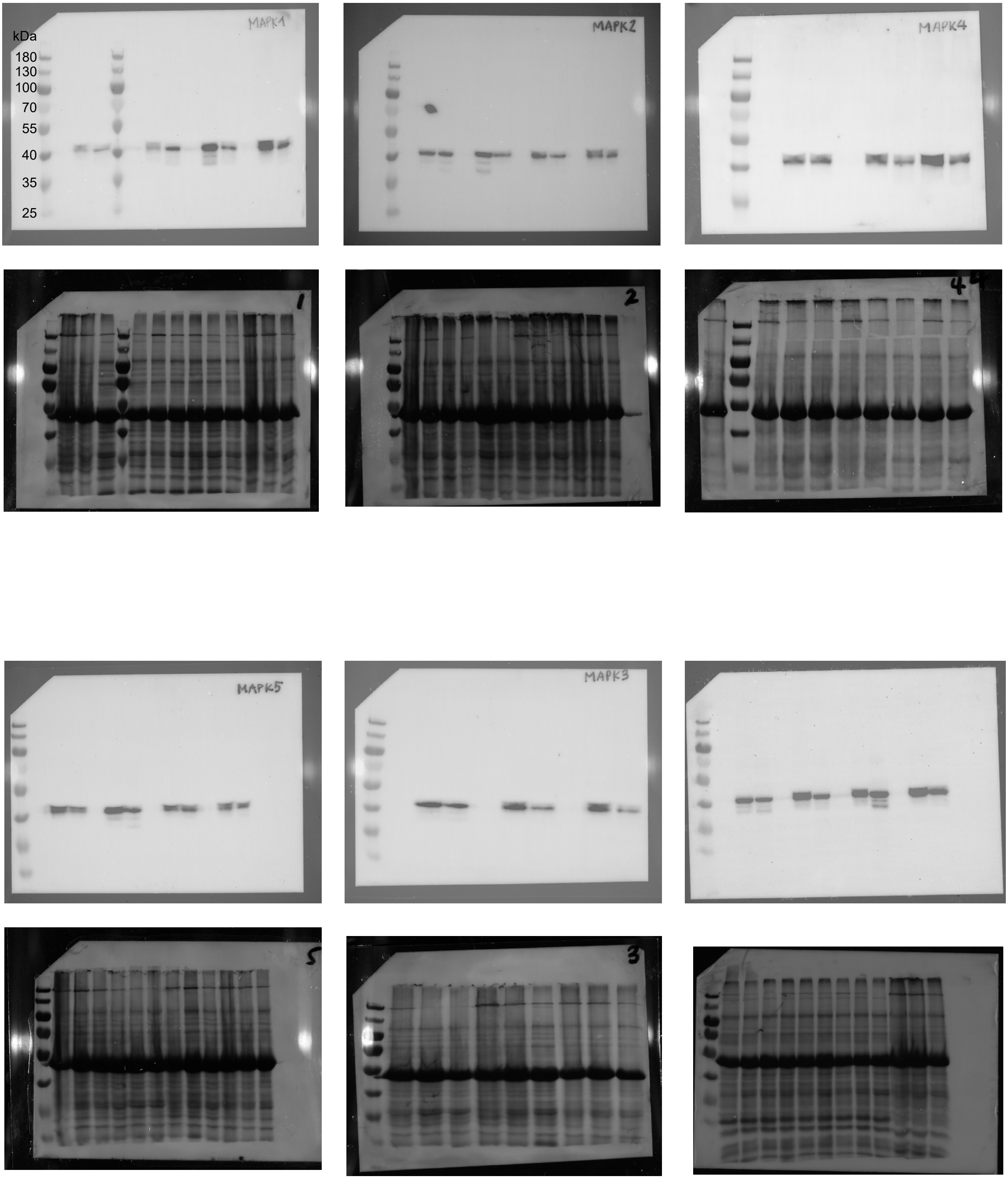

Extended Data Fig. 4

d

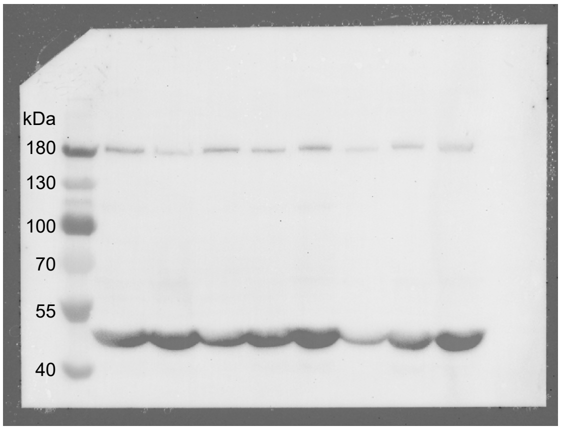

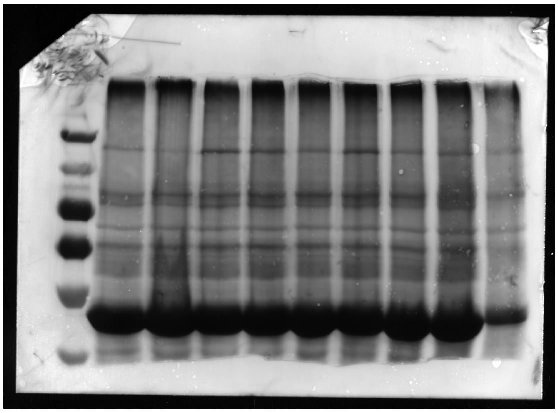

e

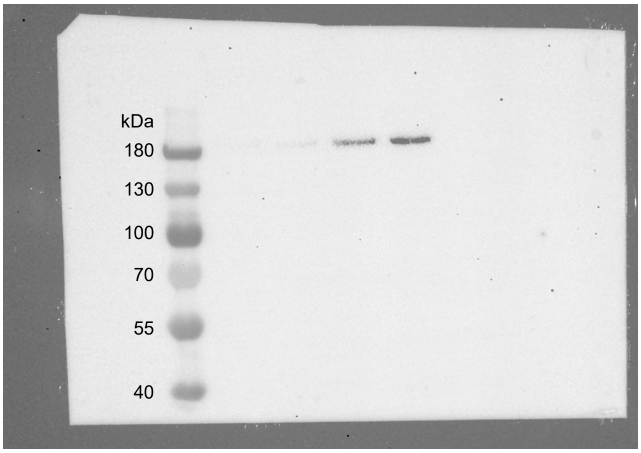

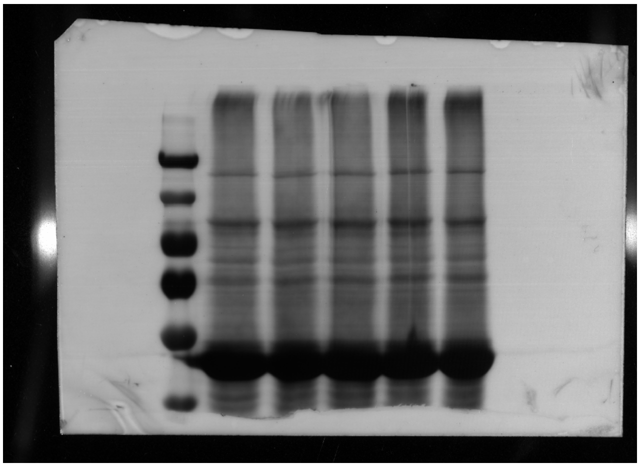

Extended Data Fig. 5g

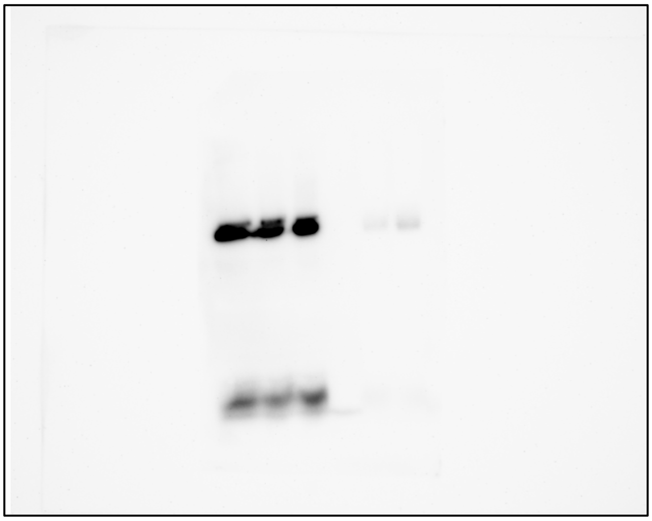

the same blot merged with molecular weight marker but with different exposure time

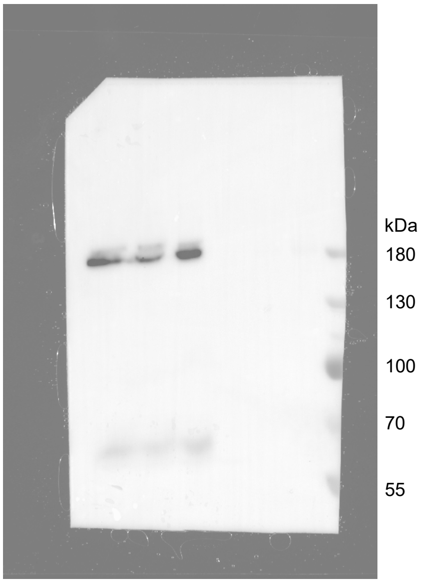

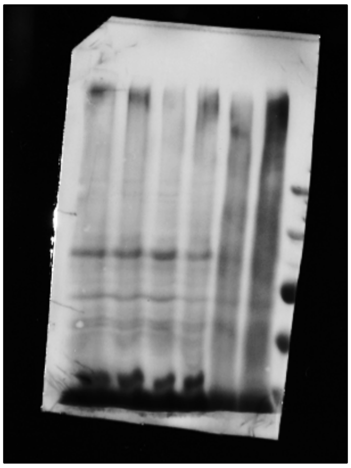

Extended Data Fig. 5i

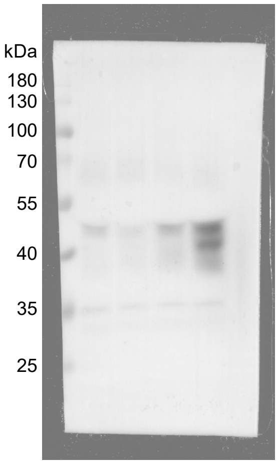

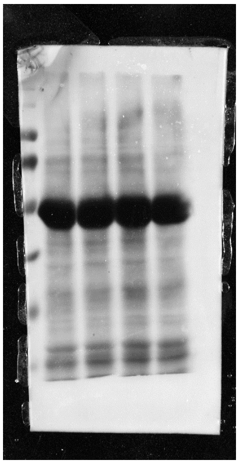

Extended Data Fig. 6g

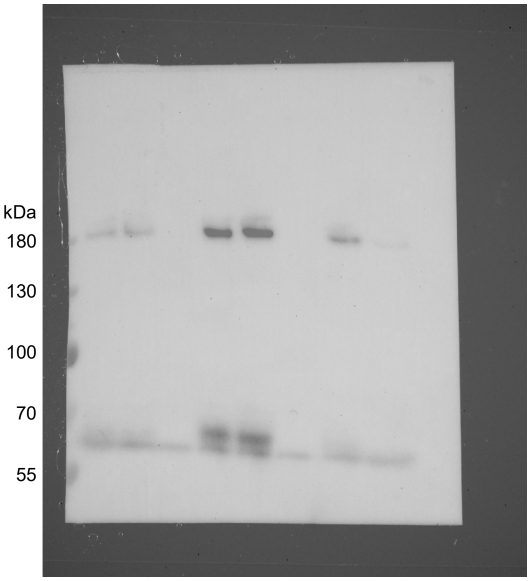

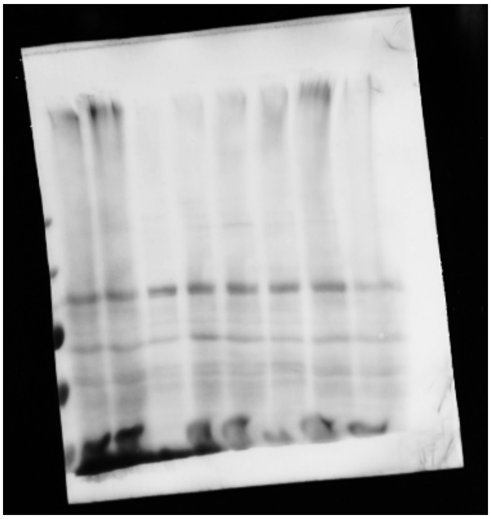

Supplement: Supplementary file 8 — Unprocessed images of western blots and histochemical GUS assay. [file 41477_2025_2050_MOESM8_ESM.pdf]
